# β-catenin-inhibited Sumoylation modification of LKB1 and fatty acid metabolism is critical in renal fibrosis

**DOI:** 10.1038/s41419-024-07154-y

**Published:** 2024-10-22

**Authors:** Shuangqin Chen, Jiemei Li, Ye Liang, Meijia Zhang, Ziqi Qiu, Sirui Liu, HaoRan Wang, Ye Zhu, Shicong Song, Xiaotao Hou, Canzhen Liu, Qinyu Wu, Mingsheng Zhu, Weiwei Shen, Jinhua Miao, Fan Fan Hou, Youhua Liu, Cheng Wang, Lili Zhou

**Affiliations:** 1 State Key Laboratory of Organ Failure Research, National Clinical Research Center of Kidney Disease, Guangdong Provincial Clinical Research Center for Kidney Disease, Guangdong Provincial Key Laboratory of Nephrology, Division of Nephrology, Nanfang Hospital, Southern Medical University, Guangzhou, China; 2 Division of Nephrology, Department of Medicine, The Fifth Affiliated Hospital of Sun Yat-Sen University, Zhuhai, China; 3 Walter Johnson High School, Bethesda, MD USA; 4 Pathology Department, Guangzhou KingMed Center for Clinical Laboratory Co., Ltd, Guangzhou, China

**Keywords:** Metabolic disorders, Chronic kidney disease, Sumoylation

## Abstract

Liver kinase B1 (LKB1) is a serine/threonine kinase controlling cell homeostasis. Among post-translational modification, Sumoylation is vital for LKB1 activating adenosine 5’-monophosphate (AMP)-activated protein kinase (AMPK), the key regulator in energy metabolism. Of note, AMPK-regulated fatty acid metabolism is highly involved in maintaining normal renal function. However, the regulative mechanisms of LKB1 Sumoylation remain elusive. In this study, we demonstrated that β-catenin, a notorious signal in renal fibrosis, inhibited the Sumoylation of LKB1, thereby disrupting fatty acid oxidation in renal tubular cells and triggering renal fibrosis. Mechanically, we found that Sumo3 was the key mediator for LKB1 Sumoylation in renal tubular cells, which was transcriptionally inhibited by β-catenin/Transcription factor 4 (TCF4) signaling. Overexpression of Sumo3, not Sumo1 or Sumo2, restored β-catenin-disrupted fatty acid metabolism, and retarded lipid accumulation and fibrogenesis in the kidney. In vivo, conditional knockout of β-catenin in tubular cells effectively preserved fatty acid oxidation and blocked lipid accumulation by maintaining LKB1 Sumoylation and AMPK activation. Furthermore, ectopic expression of Sumo3 strongly inhibited Wnt1-aggravated lipid accumulation and fibrogenesis in unilateral ischemia-reperfusion mice. In patients with chronic kidney disease, we found a loss of Sumo3 expression, and it was highly related to LKB1 repression. This contributes to fatty acid metabolism disruption and lipid accumulation, resulting in renal fibrosis. Overall, our study revealed a new mechanism in fatty acid metabolism dysfunction and provided a new therapeutic target pathway for regulating Sumo modification in renal fibrosis.

## Introduction

Chronic kidney disease (CKD), with high morbidity and mortality, is becoming a public health problem worldwide [1, 2]. As the common pathological feature of CKD, renal fibrosis severely accelerates the loss of kidney function by promoting the massive loss of nephrons [3]. Of note, current therapies for renal fibrosis still remain unsatisfactory, mostly due to undiscovered mechanisms [3–5]. Among multiple cells, tubular epithelial cells deserve superior notifications because of their primary role in kidney parenchyma [6]. Indeed, recent findings show they play a vital role in the pathogenesis of renal fibrosis. The reason behind this lies in their abnormality in metabolism in kidney injury [4, 7]. However, the underlying mechanisms of metabolism dysfunction in renal tubular cells are not elucidated.

Energy supply, from fatty acid metabolism, is fundamental for various organic cell survival and signal transduction [8, 9]. Fatty acid metabolism disorders strongly contribute to disease progression in various diseases, such as tumors, central nervous system disorders, and organ fibrosis [9–11]. Especially, fatty acid metabolism dysfunction obviously occurred in both acute and chronic stages of kidney diseases. However, the regulated mechanisms have not been found in detail.

To meet high energy consumption, renal tubular epithelial cells strongly depend on fatty acid oxidation for adenosine triphosphate (ATP) production. Abnormalities in this aspect would certainly drive a series of pathological processes to trigger renal fibrosis [4, 7, 8, 12], while the regulating mechanisms are still unclear.

AMPK, the center master controlling lipid and glucose metabolism, protein synthesis, and others, is the key factor for maintaining cell homeostasis [13, 14]. AMPK is endogenously modulated by LKB1, a serine/threonine kinase with native protein at a molecular weight of ~50 kDa [15]. LKB1 is fundamental for keeping cellular homeostasis in renal tubular cells, and gene ablation of it would trigger fibrogenesis. These suggest to modulate LKB1 would represent future directions to explore the therapeutic approaches to fight against renal fibrosis. Indeed, several studies, including ours, have found pharmacological activation or gene expression of LKB1 retard renal fibrosis [16, 17]. It is worth noting, as a kinase protein, LKB1 could be variously modified by post-translational modifications, including phosphorylation, prenylation, ubiquitination, and especially Sumoylation [15, 18]. These modifications highly correlate with the function of LKB1 in regulating downstream signals. Truly, a recent study found Sumoylated LKB1 precisely controls AMPK activation [19]. Nevertheless, the modulators controlling LKB1 Sumoylation are still in mystery.

Wnt/β-catenin is essential in regulating organ development and tissue homeostasis but is also involved in the pathogenesis of diseases. Previous studies by our groups found it is highly associated with kidney fibrotic lesions [20, 21]. In tumors, β-catenin promotes neoplasm through repressing the LKB1/AMPK pathway [22, 23]. In kidney diseases, the role of β-catenin in the LKB1 pathway has not been clarified, and the underlying mechanisms should be investigated in detail. Moreover, the regulatory role of β-catenin in fatty acid metabolism in renal tubular cells should be demonstrated clearly.

In this study, we found that the Sumoylation of LKB1 was decreased in renal fibrosis, which was highly controlled by β-catenin. Through inhibition of Sumo3 transcription, β-catenin repressed Sumoylated modification of LKB1, to further block AMPK activity and fatty acid metabolism. These led to the fibrogenesis in renal tubular cells and the progression of renal fibrosis. Supplementation of Sumo3 preserved fatty acid metabolism, inhibited lipid accumulation, and retarded renal fibrosis. Our study provided a new mechanism of renal fibrosis, and provided a potential therapeutic approach of regulating Sumoylation modification to treat renal fibrosis.

## Results

### Sumoylation, LKB1-AMPK signaling, and fatty acid metabolism were inhibited in UIRI mice

**Fig. 1:**
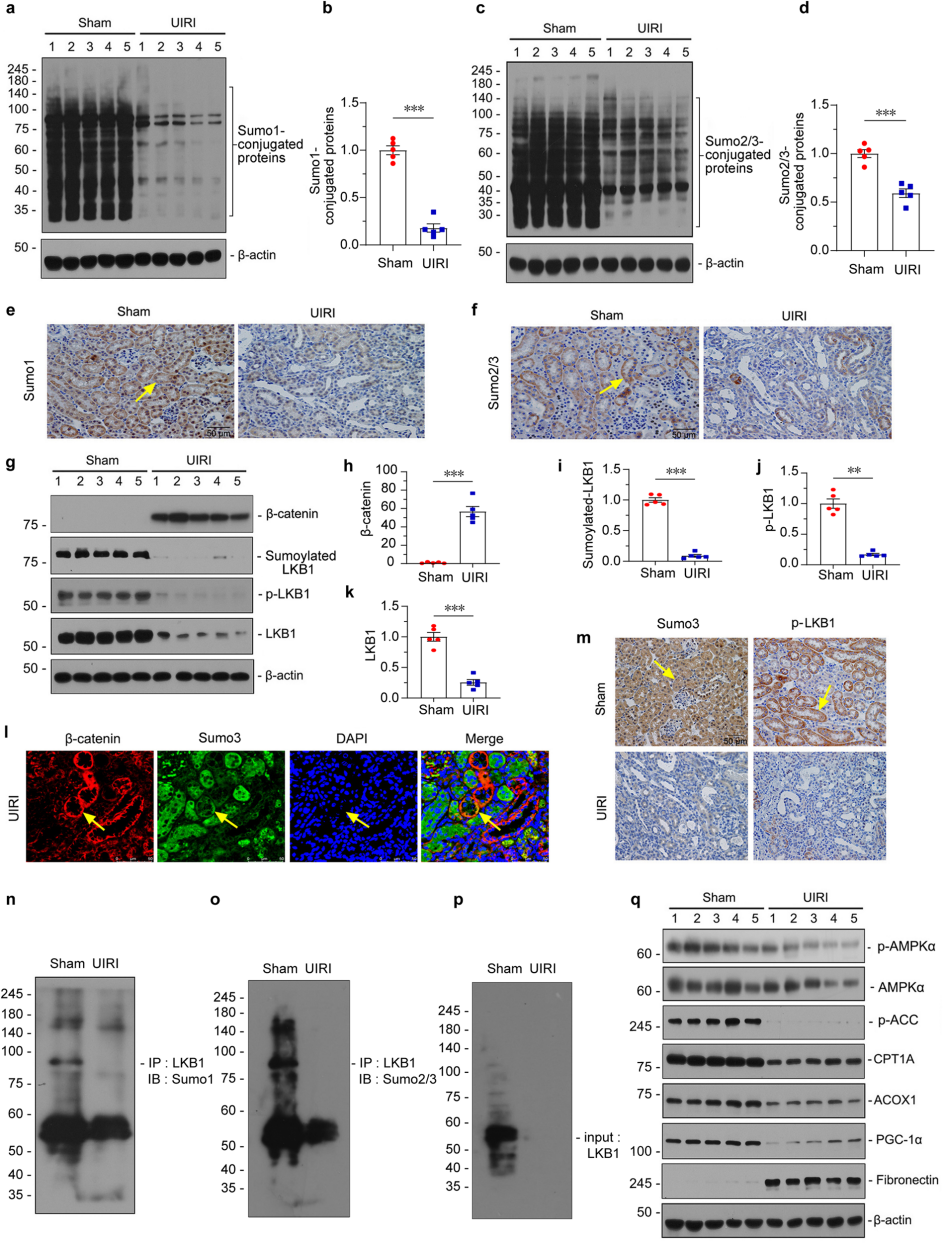
Sumoylation, LKB1-AMPK signaling, and fatty acid metabolism were inhibited in UIRI. **a** and **b** Representative western blotting and quantitative data showing renal expression of Sumo1-conjugated proteins in unilateral ischemia-reperfusion injury (UIRI) and sham mice. UIRI mice were sacrificed at 11 days after ischemia-reperfusion surgery. Numbers (1–5) indicate individual animals in a given group. ***P < 0.001 versus sham controls (*n* = 5). **c** and **d** Representative western blotting and quantitative data showing renal expression of Sumo2/3-conjugated proteins in UIRI and sham mice. Numbers (1–5) indicate individual animals in a given group. ***P < 0.001 versus sham controls (*n* = 5). **e** and **f** Representative micrographs of immunohistochemical staining showing Sumo1 and Sumo2/3 expression in UIRI mice. Kidney paraffin sections were stained with antibodies against Sumo1 or Sumo2/3. Arrows indicate positive staining; scale bar: 50 µm. **g**–**k** Western blotting and quantitative data showing renal expression of β-catenin, Sumoylated-LKB1, p-LKB1 and LKB1 in UIRI and sham mice. Numbers (1–5) indicate individual animals in a given group. **P < 0.01, ***P < 0.001 versus sham controls (*n* = 5). **l** Immunofluorescence staining showing staggered expression of Sumo3 and β-catenin in UIRI mice. Kidney paraffin sections were stained for β-catenin (red) and Sumo3 (green). Arrows indicate β-catenin positive tubules with a lost expression of Sumo3; scale bar: 50 µm. **m** Representative micrographs showing immunohistochemical staining of Sumo3 (left) and p-LKB1 (right) in UIRI mice. Kidney paraffin sections were stained with antibodies against Sumo3 or p-LKB1. Arrows indicate positive staining; scale bar: 50 µm. **n–p** Immunoprecipitation showing the binding of LKB1 with Sumo1 or Sumo2/3 in kidney tissue from UIRI mice and Sham controls. The whole cell lysates were immunoprecipitated (IP) with anti-LKB1, followed by immunoblotting (IB) with anti-Sumo2/3, or anti-Sumo1, respectively. Total diluted lysates were used as input. **q** Western blotting showing renal expression of p-AMPKα/AMPKα, p-ACC, CPT1A, ACOX1, PGC-1α and Fibronectin in UIRI and sham mice. Numbers (1–5) indicate individual animals in a given group. The data were analyzed by using Student’s *t*-test.

We first established a CKD mice model through performing unilateral ischemia/reperfusion (UIRI) surgery. To testify the condition of Sumoylation, Sumo1 and Sumo2/3-conjugated proteins were examined by western blotting. As shown in Fig. 1a–d, compared to sham mice, both Sumo1 and Sumo2/3-conjugated proteins were strongly downregulated in UIRI mice. Similar results were observed when Sumo1 and Sumo2/3 were assessed by immunohistochemistry staining (Fig. 1e, f), and qRT-PCR assessment (Supplementary Fig. S1). Additionally, we also found that E1 Sumo-activating enzymes (SAE1/SAE2), a single E2 conjugating enzyme (UBC9), and a set of E3 Sumo ligases were all downregulated in UIRI mice (Supplementary Fig. S1).

LKB1 is a master regulator for energy metabolism. The modification of Sumoylation and phosphorylation is vital for the active activity of LKB1. As shown in Fig. 1g–j, in UIRI mice, both Sumoylation and phosphorylation of LKB1 were greatly blocked, accompanied by the decrease in native LKB1, and this was concomitant with a high increase in β-catenin, suggesting the intimate relationship between β-catenin and LKB1 Sumoylation and activation. Of interest, we observed a staggered expression of β-catenin and Sumo3 in UIRI mice (Fig. 1l). More importantly, we found Sumo3 and p-LKB1 were both highly expressed in Sham control mice, but were both greatly lost in UIRI mice (Fig. 1m). We then performed immunoprecipitation assay to testify the Sumoylated LKB1. As shown in Fig. 1n–p, Sumo1-modified LKB1 was decreased in UIRI mice. However, the Sumoylation of LKB1 by Sumo2/3 was inhibited more pronouncedly.

AMPK signaling and fatty acid oxidation (FAO) are masterly regulated by LKB1. We then assessed them in UIRI mice. As shown in Fig. 1q and Supplementary Fig. S1, p-AMPKα, AMPKα, phosphorylated acetyl-CoA carboxylase (p-ACC) and a variety of FAO-related proteins such as carnitine palmitoyl-transferase 1A (CPT1A), acyl-CoA oxidase 1 (ACOX1) and peroxisome proliferator-activated receptor gamma coactivator-1 alpha (PGC-1α) were reduced in UIRI mice, accompanied by the increase in Fibronectin expression and lipid accumulation (Supplementary Fig. S1). These results suggested that the downregulation of LKB1 Sumoylation was highly associated with lipid metabolism abnormality in the kidney and contributed to renal fibrosis. Furthermore, β-catenin was possibly the important upstream modulator.

### β-catenin inhibits AMPK signaling and fatty acid metabolism by blocking LKB1 Sumoylation

**Fig. 2:**
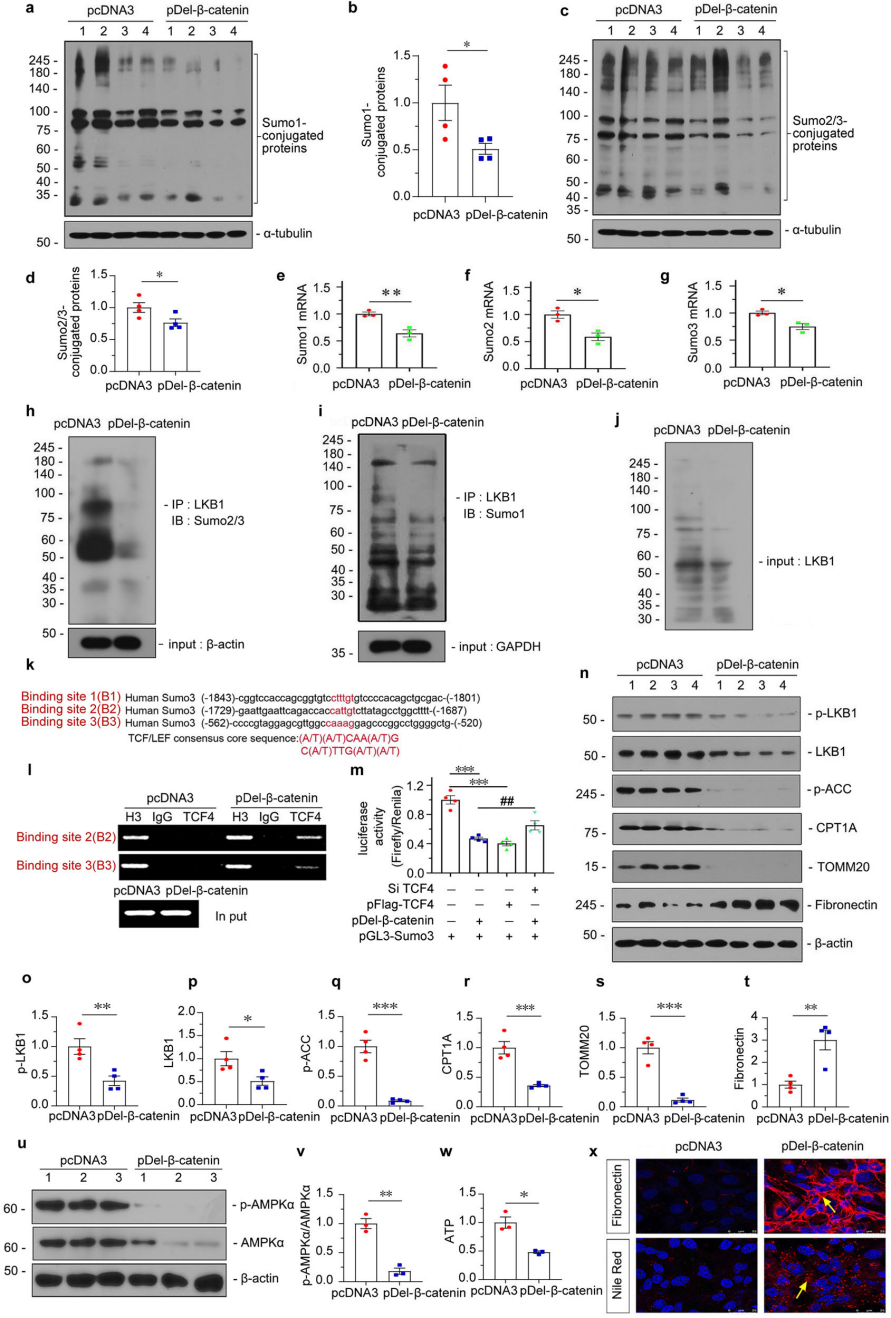
β-catenin inhibits AMPK signaling and fatty acid metabolism by blocking LKB1 Sumoylation. Human kidney proximal tubular epithelial cells (HKC-8) were transfected with β-catenin expression plasmid (pDel-β-catenin) or pcDNA3 for 24 h. **a** and **b** Western blotting and quantitative data showing protein expression of Sumo1-conjugated proteins in different groups. Numbers (1–4) indicate individual culture in a given group. *P < 0.05 versus pcDNA3 (*n* = 4). **c** and **d** Representative western blotting and quantitative data for Sumo2/3-conjugated proteins are shown. *P < 0.05 versus pcDNA3 (*n* = 4). **e–g** Quantitative real-time PCR analyses showing mRNA expression of Sumo1, Sumo2 and Sumo3 in different groups. *P < 0.05, **P < 0.01 versus pcDNA3 (*n* = 3). **h–j** Immunoprecipitation showing the binding of LKB1 with Sumo1 or Sumo2/3 in HKC-8 cells transfected with pcDNA3 or pDel-β-catenin plasmid. Cell lysates were immunoprecipitated (IP) with anti-LKB1, followed by immunoblotted (IB) with anti-Sumo2/3, or anti-Sumo1, respectively. Total diluted lysates were used as input. **k** and **l** Bioinformation analysis and Chromatin immunoprecipitation (ChIP) assay revealing the presence of TCF/LEF binding sites in the promoter region of the Sumo3 gene. The sequences and positions of the putative sites were shown, and the consensus sequence was also given. HKC-8 cells were transfected with pcDNA3 or pDel-β-catenin plasmid for 24 h. Cell DNAs were precipitated with anti-H3, nonimmune IgG, and anti-TCF4. Total DNA lysates were used as input. The PCR assay was performed to detect specific binding consensus sequences in the Sumo3 gene promoter region. Total diluted lysate was used as the total genomic input DNA. **m** Quantitative data showing the luciferase activity of pGL3-Sumo3 (constructed with the full length of Sumo3 gene promoter sequence) in HKC-8 cells co-transfected with pDel-β-catenin, pFlag-TCF4, or pDel-β-catenin and TCF4 siRNA for 24 h. Renilla was added as an internal control reporter. ***P < 0.001 versus pGL3-Sumo3 group, ^##^*P* < 0.01 versus pDel-β-catenin + pGL3-Sumo3 group (*n* = 4). **n–t** Representative western blotting and quantitative data showing protein expression of p-LKB1, LKB1, p-ACC, CPT1A, TOMM20 and Fibronectin in different groups. HKC-8 cells were transfected with pDel-β-catenin or pcDNA3 for 24 h. Numbers (1–4) indicate individual culture in a given group. *P < 0.05, **P < 0.01, ***P < 0.001 versus pcDNA3 (*n* = 4). **u** and **v** Representative western blotting and quantitative data showing the ratio of p-AMPKα/AMPKα. Numbers (1–3) indicate individual culture in a given group. **P < 0.01 versus pcDNA3. **w** Quantitative data showing ATP levels in different groups. *P < 0.05 versus pcDNA3 (*n* = 3). **x** Representative micrographs showing immunofluorescence staining of Fibronectin (upper) and Nile Red (bottom). Arrows indicate positive staining; scale bar: 25 µm. The data were analyzed by using Student’s *t*-test or one-way ANOVA.

To clearly elucidate the relationship between β-catenin and LKB1 Sumoylation, HKC-8 cells, a human proximal tubular cell line, were transfected with β-catenin expression plasmid (pDel-β-catenin). The efficacy of β-catenin overexpression was first confirmed (Supplementary Fig. S2). As shown in Fig. 2a–d, β-catenin overexpression reduced the expression of Sumo1 and Sumo2/3-conjugated proteins. We further found that β-catenin also decreased the mRNA levels of Sumo1, Sumo2, and Sumo3 (Fig. 2e–g).

The Sumoylation of LKB1 was then examined by immunoprecipitation assay. As shown in Fig. 2h–j, both Sumo1 and Sumo2/3-modified LKB1 were inhibited by ectopic β-catenin, especially Sumo2/3-conjugated LKB1 decreased more greatly. We then analyzed the human Sumo genes promoter region and found that the Sumo3 promoter possesses three predictive binding sites of TCF/LEF (Fig. 2k), the downstream transcription factors of β-catenin, while Sumo2 possesses 2 and Sumo1 possesses 1 (data not shown). ChIP assay was then conducted to determine the specificity of Sumo3 binding with TCF4. As shown, 2 predicted binding sites (B2 and B3) in the Sumo3 gene promoter were identified specifically for binding with TCF4 (Fig. 2l). Consistently, luciferase assay confirmed β-catenin or TCF4 transcriptionally inhibited Sumo3, while interference of TCF4 blocked the depression of β-catenin on Sumo3 transcription (Fig. 2m).

Next, LKB1-AMPK signaling and fatty acid metabolism were assessed in β-catenin-overexpressed cells. As shown in Fig. 2n–w, β-catenin inhibited p-LKB1, LKB1, p-AMPKα, AMPKα, p-ACC, CPT1A and TOMM20. Fibrosis and lipid accumulation were also tested. As shown, β-catenin triggered Fibronectin expression and the accumulation of lipid droplets, as well as reduced ATP production (Fig. 2n, t, w, x). These results further suggested β-catenin negatively regulates LKB1 Sumoylation through inhibiting Sumo3.

### Sumo3 plays a decisive role in Sumoylation modification of LKB1

**Fig. 3:**
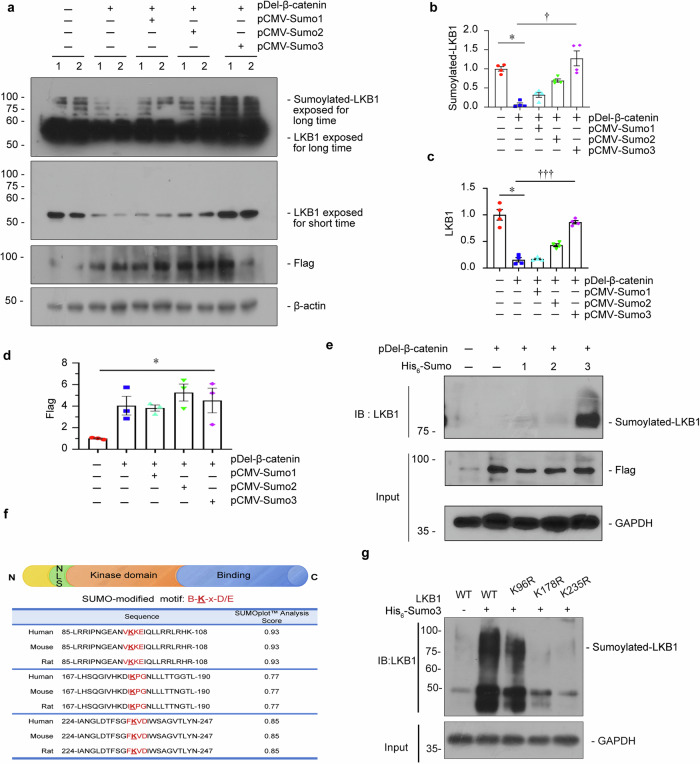
Sumo3 plays a decisive role in Sumoylation modification of LKB1. **a–d** Representative western blotting and quantitative data showing protein expression of Sumoylated-LKB1, LKB1 and Flag in different groups. HKC-8 cells were transfected with pDel-β-catenin and Sumo1 expression plasmid (pCMV-Sumo1), Sumo2 expression plasmid (pCMV-Sumo2) or Sumo3 expression plasmid (pCMV-Sumo3) for 24 h. Numbers (1–2) indicate individual culture in a given group. *P < 0.05 versus pcDNA3; ^**†**^*P* < 0.05, ^**†††**^*P* <0.001 versus pDel-β-catenin. **e** Ni-NTA MagBeads-pulldown assay showing Sumo3 restored the Sumoylated modification of LKB1 in HKC8 cells. HKC8 cells were transfected with the His_6_-Sumo1, 2, 3 plasmids with or without pDel-β-catenin. The His_6_-Sumolyated proteins were purified with HisSep Ni-NTA MagBeads, followed by IB with anti-LKB1. The whole lysates were used as input. **f** Graphs showing the full structure of LKB1 and the predicted Sumoylated residues of LKB1 in different species. **g** Ni-NTA MagBeads-pulldown assay showing LKB1 was mostly Sumoylated by Sumo3 at Lys178 and Lys235. HKC8 cells were transfected with His_6_-Sumo3 plasmid (or pcDNA3), simultaneously with LKB1 mutant plasmids or wildtype LKB1 expression plasmid. The His_6_-Sumolyated proteins were purified with HisSep Ni-NTA MagBeads, followed by IB with anti-LKB1. The whole lysates were used as input. The data were analyzed by using one-way ANOVA.

To clarify which Sumo decides Sumoylation modification of LKB1, HKC-8 cells were transfected with pDel-β-catenin plasmid and co-treated with Sumo1, 2 and 3 expression plasmids (pCMV-Sumo1–3), respectively. The efficiencies of β-catenin, Sumo1, Sumo2 and Sumo3 overexpression were confirmed by western blotting and immunofluorescence (Fig. 3a, d, and Supplementary Fig. S2). As shown, β-catenin inhibited LKB1 Sumoylation and LKB1 expression, but co-treatment with Sumo3 extremely restored Sumoylated LKB1 and also preserved native LKB1 expression, while Sumo1 and Sumo2 did not show the same effects (Fig. 3a–c).

In order to identify the Sumoylation and Sumo-bingding sites of LKB1 in renal tubular cells, Ni-NTA MagBeads-pulldown assay was performed. HKC-8 cells were co-transfected with pDel-β-catenin and the His_6_-Sumo1, 2, or 3 plasmids. As shown, Sumo3, but not Sumo1 or Sumo2, greatly restored the Sumoylated modification of LKB1 (Fig. 3e). By using SUMOplot^TM^ (http://www.abgent.com/sumoplot), we predicted the Sumoylation residues in LKB1, and found 3 highly conserved residues (K96, K178, and K235) for LKB1 Sumoylation (Fig. 3f). We then constructed LKB1 wild-type plasmid and mutated plasmids with the above residue (lysine) mutating to arginine. As shown in Fig. 3g, Sumo3 interacted with wild-type LKB1, while mutation on K178 and K235 abandoned Sumo3-conjugated Sumoylation, suggesting Lys178 and Lys235 were the key binding sites of Sumo conjugating to LKB1.

### Sumo3 preserved FAO metabolism and inhibited fibrogenesis in cultured tubular cells

**Fig. 4:**
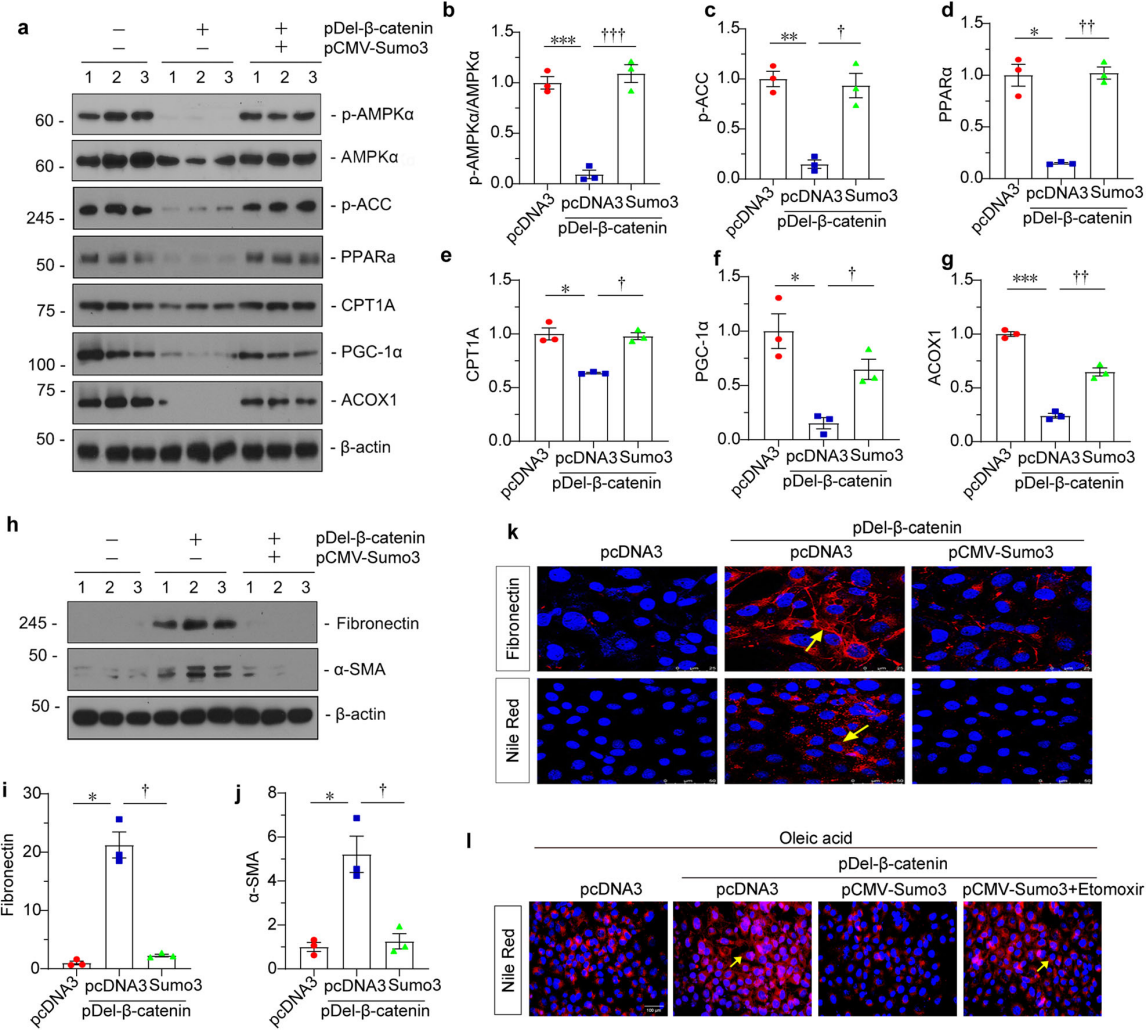
Sumo3 preserved FAO metabolism and inhibited fibrogenesis in cultured tubular cells. **a–g** Representative western blotting and quantitative data showing protein expression of p-AMPKα/AMPKα, p-ACC, PPARα, CPT1A, PGC-1α and ACOX1 in different groups. HKC-8 cells were transfected with pDel-β-catenin alone or with pCMV-Sumo3 for 24 h. Numbers (1–3) indicate individual culture in a given group. *P < 0.05, **P < 0.01, ***P < 0.001 versus pcDNA3; ^**†**^*P* < 0.05, ^**††**^*P* <0.01, ^**†††**^*P* < 0.001 versus pDel-β-catenin (*n* = 3). **h–j** Representative western blotting and quantitative data showing protein expression of Fibronectin and α-SMA in different groups. Numbers (1–3) indicate individual culture in a given group. *P < 0.05 versus pcDNA3; ^**†**^*P* < 0.05 versus pDel-β-catenin (*n* = 3). **k** Representative micrographs showing immunofluorescence staining of Fibronectin (upper) and Nile Red (bottom) in different groups. Arrows indicate positive staining. For Fibronectin staining, scale bar: 25 µm. For Nile red staining, scale bar: 50 µm. **l** Representative immunofluorescence micrographs showing the expression of lipid (Nile Red) in different groups. Arrows indicate positive staining; scale bar, 100 µm. HKC-8 cells were transfected with pDel‐β‐catenin alone or with pCMV-Sumo3, or co-treated with etomoxir (40 μM, HY-50202; MCE) for 18 h, and then all added with sodium salt form of oleic acid (200 μM, HY-N1446B; MCE) for 24 h. The data were analyzed by one-way ANOVA.

We then assessed FAO metabolism and fibrogenesis. As shown, p-AMPKα, AMPKα, p-ACC, peroxisome proliferator-activated receptor alpha (PPARα), CPT1A, PGC-1α, and ACOX1 were significantly downregulated in β-catenin-overexpressed tubular cells (Fig. 4a–g). However, co-treatment with Sumo3 restored all these proteins' expression. A similar result was observed when ATP production was examined (Supplementary Fig. S2). Furthermore, fibrogenesis and lipid accumulation were assessed. As shown, Sumo3 ameliorated β-catenin-induced Fibronectin and α-SMA upregulation and lipid accumulation (Fig. 4h–k). To further test fatty acid metabolism, HKC-8 cells were administered oleic acid [24], and assessed lipid droplets by Nile red staining. As shown, β-catenin exaggerated lipid deposition, while co-treatment with Sumo3 blocked it. Furthermore, Etomoxir, an inhibitor to CPT1A, abolished the effects of Sumo3 in β-catenin-overexpressed cells (Fig. 4l). Some cells were also treated with TGF-β1, a potent fibrogenic factor. As shown in Supplementary Fig. S2, ectopic Sumo3 also restored LKB1-AMPK signaling and FAO function and inhibited fibronectin expression in TGF-β1-treated cells.

**Fig. 5:**
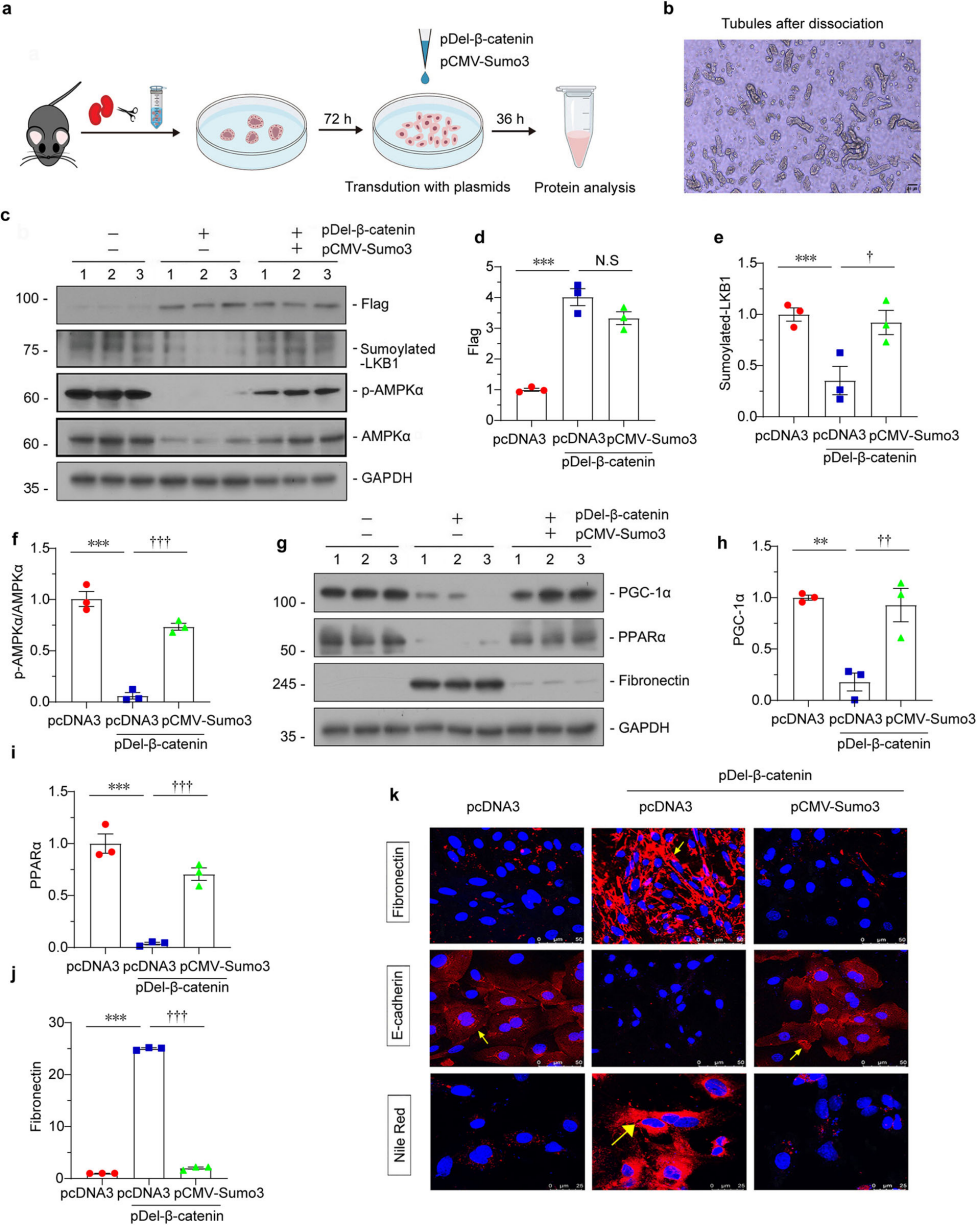
Sumo3 plays a key role in LKB1 Sumoylation and keeping FAO function in primary cultured renal tubular epithelial cells. **a** and **b** Experimental design. Primary renal tubular epithelial cells were isolated from C57BL/6 mice following a routine protocol. The kidneys were minced, digested, filtrated and centrifuged. Then, the tubules were resuspended in DMEM/F12 medium with 10% FBS and seeded in a six-well plate. After growing for 72 h, the cells were transfected with pDel-β-catenin alone or co-transfected with pCMV-Sumo3 for 36 h. Dissociated tubules are shown. Scale bar: 50 µm. **c–f** Representative western blotting and quantitative data showing protein expression of Flag, Sumoylated-LKB1 and p-AMPKα/AMPKα in different groups. ***P < 0.001 versus pcDNA3; ^**†**^*P* < 0.05, ^**†††**^*P* < 0.001 versus pDel-β-catenin, N.S.: none of significance. (*n* = 3). **g–j** Representative western blotting and quantitative data showing protein expression of PGC-1α, PPARα and Fibronectin in different groups. **P < 0.01, ***P < 0.001 versus pcDNA3; ^**††**^*P* < 0.01, ^**†††**^*P* < 0.001 versus pDel-β-catenin (*n* = 3). **k** Representative immunofluorescence staining of Fibronectin (upper), E-cadherin (middle) and Nile Red (bottom) in different groups. Arrows indicate positive staining; For Fibronectin or E-cadherin, scale bar: 50 µm. For Nile Red staining, scale bar: 25 µm. The data were analyzed by using one-way ANOVA.

We next isolated and cultured primary renal tubular cells (Fig. 5a, b). These cells were transfected with pDel-β-catenin plasmid or co-treated with Sumo3 expression plasmid. The efficacy of β-catenin overexpression was confirmed by western blotting (Fig. 5c, d). We found Sumoylated LKB1 was restored by Sumo3. Consequently, p-AMPKα, AMPKα, PGC-1α and PPARα expression levels were all preserved by Sumo3, while Fibronectin was inhibited by Sumo3 (Fig. 5c–j). Similar results were observed when Fibronectin was assessed by immunofluorescence (Fig. 5k). Furthermore, immunostaining and Nile red staining results showed Sumo3 preserved E-cadherin, a marker for tubular cell integrity, and inhibited lipid deposition in β-catenin-overexpressed primary cells (Fig. 5k).

### Knockout of β-catenin increased fatty acids metabolism in UUO mice

**Fig. 6:**
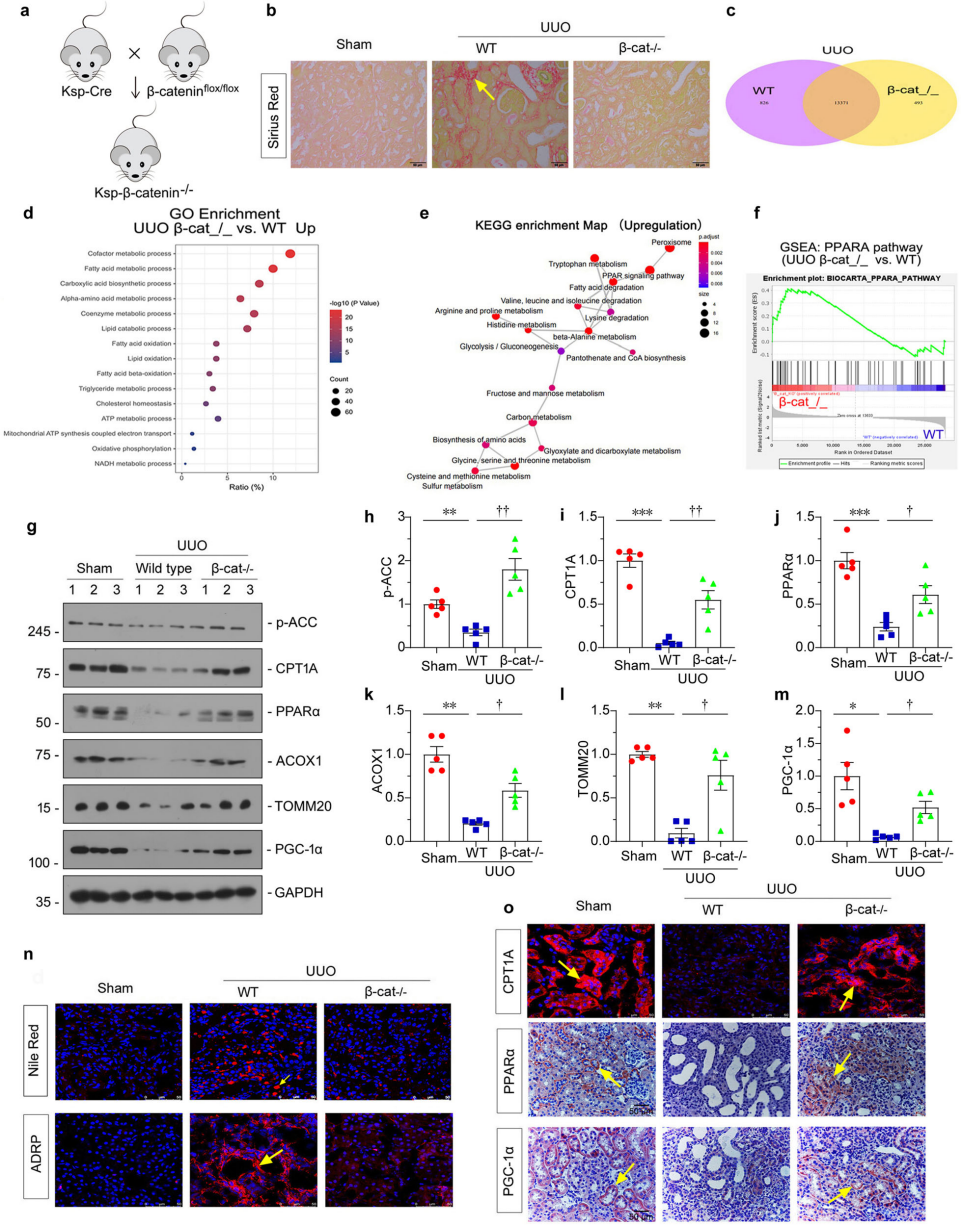
Knockout of β-catenin increased fatty acids metabolism in UUO mice. **a** Experimental design showing the strategy of crossbreeding of the β-catenin-floxed mice (β-catenin^flox/flox^) with Cre transgenic mice under the control of the Ksp-cadherin promoter (Ksp-Cre). **b** Representative micrographs showing collagen deposition assessed by Sirius red staining in different groups. Arrow indicates positive staining; scale bar: 50 µm. **c** Graph showing the number of differentially expressed genes in UUO-affected β-catenin knockout and wild-type mice. **d** Gene ontology (GO) analysis showing the biological process enrichment of upregulated genes from UUO-affected β-catenin knockout mice. **e** Kyoto Encyclopedia of Genes and Genomes (KEGG) analysis showing multiple metabolism pathways were enriched in the UUO + β-catenin^−/−^ group. **f** Gene set enrichment analysis (GSEA) showing the PPARα pathway was up-regulated in the UUO + β-catenin^−/−^ group. **g–m** Representative western blotting and quantitative data showing protein expression of p-ACC, CPT1A, PPARα, ACOX1, TOMM20 and PGC-1α in different groups. Numbers (1–3) indicate individual animals in a given group. *P < 0.05, **P < 0.01, ***P < 0.001 versus sham controls; ^**†**^*P* < 0.05, ^**††**^*P* < 0.01 versus WT UUO mice (*n* = 5). **n** Representative immunofluorescence staining of Nile Red (upper) and ADRP (bottom) in different groups. Kidney frozen sections were stained for ADRP expression and lipid accumulation (Nile Red). Arrows indicate positive staining; scale bar: 50 µm. **o** Representative immunohistochemical staining of CPT1A (upper), PPARα (middle) and PGC-1α (bottom). Frozen sections were stained with an antibody against CPT1A. Paraffin sections were stained with antibodies against PPARα or PGC-1α. Arrows indicate positive staining; scale bar: 50 µm. The data were analyzed by using one-way ANOVA.

Tubular cell-specific knockout of β-catenin gene (Ksp-β-cat^−/−^) mice were constructed using Cre-LoxP system (Fig. 6a) as described previously [25]. The efficacy of β-catenin gene ablation was testified by co-staining of β-catenin with LTL (lotus tetragonolobus lectin, detecting proximal tubules), PNA (peanut agglutinin, detecting distal tubules), and DBA (Dolichos biflorus agglutininm, detecting collecting duct) in Ksp-β-cat^−/−^ mice. As shown, the KSP-cre-mediated deletion of β-catenin led to β-catenin ablation in distal tubules, connecting tubules, as well as the most majority of proximal tubules (Supplementary Fig. S3).

We first assessed fibrotic lesions through Sirius red staining. As shown, unilateral ureteral obstruction (UUO) surgery resulted in matrix deposition in the interstitium, but knockout of β-catenin largely blocked it (Fig. 6b and Supplementary Fig. S3). We then performed transcriptomic sequencing to explore the gene expression differences between the two groups. Of these, 826 genes were down-regulated, and 493 genes were up-regulated in β-catenin knockout mice (Fig. 6c). GO and KEGG enrichment analysis showed β-catenin gene ablation was positively related to multiple metabolic processes, such as fatty acid metabolism, fatty acid oxidation, fatty acid β-oxidation, ATP metabolism, mitochondrial ATP synthesis and electron transport, etc (Fig. 6d, e). Gene set enrichment analysis (GSEA) also demonstrated the PPARα pathway, an important pathway for energy metabolism, was upregulated in β-catenin knockout mice (Fig. 6f).

We further assessed FAO-related protein expression. As shown in Fig. 6g–m, p-ACC, CPT1A, PPARα, ACOX1, TOMM20, and PGC-1α protein levels were downregulated in UUO mice, but they were greatly restored when β-catenin was knockout. Nile red and adipose differentiation-related protein (ADRP) staining showed lipid accumulation in UUO mice, while β-catenin gene ablation blocked it (Fig. 6n). Consistently, immunostaining also showed CPT1A, PPARα, and PGC-1α, the key regulators for fatty acid metabolism, was also preserved in β-catenin knockout mice (Fig. 6o). Similar result was observed when ATP level was assessed (Supplementary Fig. S3).

### Ablation of β-catenin restores Sumoylation of LKB1 in UUO model

**Fig. 7:**
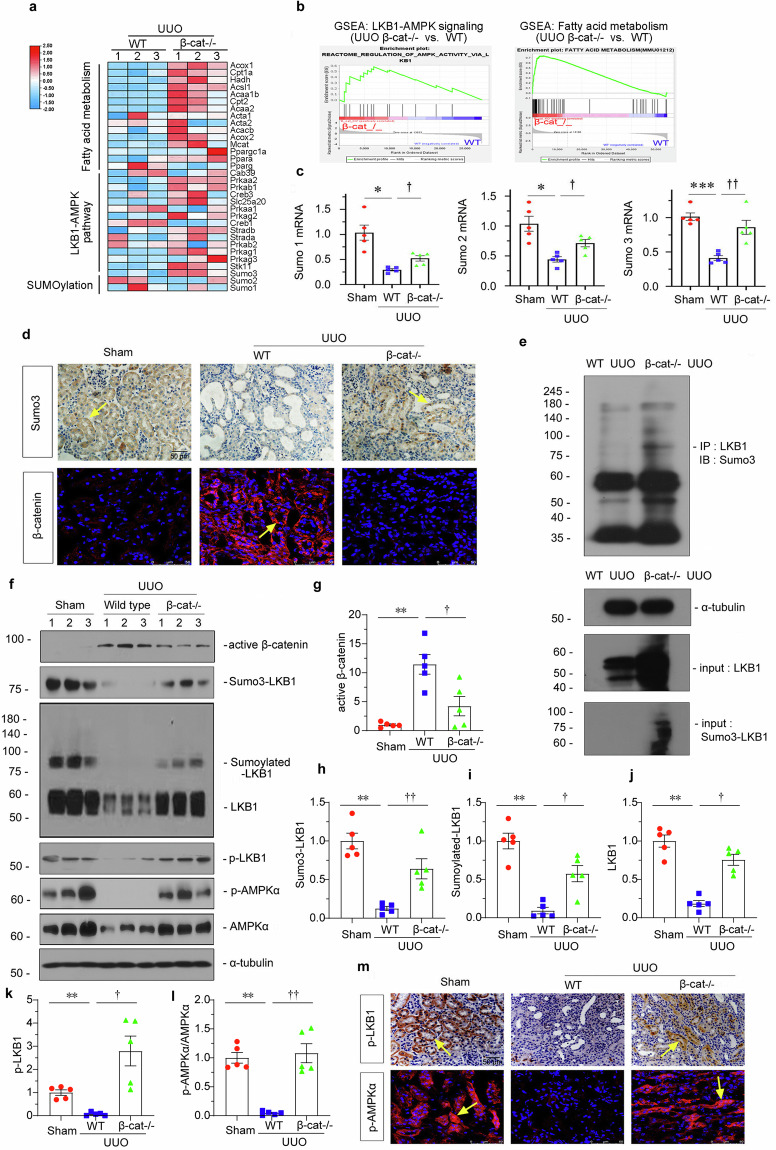
Ablation ofβ-catenin restores Sumoylation of LKB1 in UUO model. **a** Gene expression profiling by RNA-seq showing differential gene clustering of kidney from different groups. Numbers (1–3) indicate each individual animal in a given group. **b** GSEA analysis showing the LKB1-AMPK signaling and fatty acid metabolism increased in β-catenin^−/−^ UUO group compared with wildtype UUO group. **c** Quantitative real-time PCR analyses showing mRNA levels of Sumo1, Sumo2 and Sumo3 in different groups. *P < 0.05, ***P < 0.001 versus sham controls; ^**†**^*P* < 0.05, ^**††**^*P* < 0.01 versus WT UUO group (*n* = 5). **d** Representative micrographs showing renal expression of Sumo3 (upper) and β-catenin (bottom) in different groups. Paraffin and frozen sections of mice kidneys were stained with antibodies against Sumo3 or β-catenin, respectively. Arrows indicate positive staining; scale bar: 50 µm. **e** Immunoprecipitation showing the binding of Sumo3 to LKB1 in β-catenin^−/−^ UUO group or wildtype UUO group. Cell lysates were IP with anti-LKB1, followed by IB with anti-Sumo3. Total diluted lysates were used as input. **f–l** Representative western blotting and quantitative data showing the expression of active β-catenin, Sumo3-LKB1, Sumoylated-LKB1, LKB1, p-LKB1, and p-AMPKα/AMPKα in different groups. **P < 0.01 versus sham controls; ^**†**^*P* < 0.05, ^**††**^*P* < 0.01 versus WT UUO mice (*n* = 5). **m** Representative micrographs showing immunohistochemical and immunofluorescence staining of p-LKB1 (upper) and p-AMPKα (bottom). Paraffin and frozen sections were stained with antibodies against p-LKB1 or p-AMPKα. Arrows indicate positive staining; scale bar: 50 µm. The data were analyzed by using one-way ANOVA.

Through transcriptome profiling, we found LKB1-AMPK pathway and fatty acid metabolism, and also Sumo3 expression, were upregulated in β-catenin knockout mice (Fig. 7a). GSEA analysis also demonstrated gene ablation of β-catenin upregulated LKB1-AMPK signaling and enhanced fatty acid metabolism (Fig. 7b). Sumo genes expression was also assessed by qPCR analysis. As shown in Fig. 7c, Sumo1–3 mRNA levels, especially Sumo3, were all significantly decreased in UUO mice, but restored by β-catenin gene ablation. Furthermore, immunostaining was used for the detection of Sumo3 and β-catenin (Fig. 7d). As shown, Sumo3 expression decreased in UUO mice, while β-catenin knockout preserved it. We also assessed Sumo ligases by qPCR and found β-catenin ablation restored all those genes' expression (Supplementary Fig. S3).

The binding of Sumo3 with LKB1 in β-catenin knockout mice was further demonstrated by immunoprecipitation (Fig. 7e). Furthermore, western blotting was adopted to demonstrate LKB1 Sumoylation and LKB1-AMPK signals. As shown, UUO surgery inhibited Sumo3-LKB1, LKB1 Sumoylation, p-LKB1, LKB1, p-AMPKα, and AMPKα protein levels, however, β-catenin knockout mice greatly preserved them (Fig. 7f–l). Similar results were observed when p-LKB1 and p-AMPKα were assessed by immunostaining (Fig. 7m). These results further demonstrated that β-catenin inhibits LKB1 Sumoylation.

### Sumo3-mediated LKB1 Sumoylation alleviates renal fibrosis through upregulating fatty acid metabolism

**Fig. 8:**
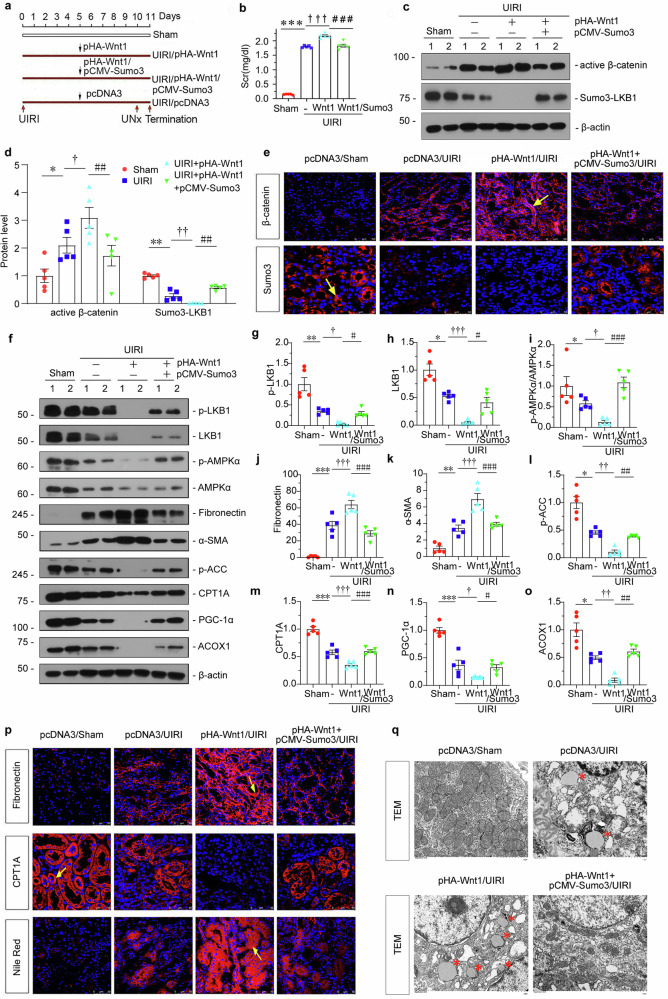
Sumo3-mediated LKB1 Sumoylation alleviates renal fibrosis through upregulating fatty acid metabolism. **a** Diagram showing the experimental design. Black arrows indicate intravenous injections of pcDNA3, Wnt1 overexpression plasmid (pHA-Wnt1) or Sumo3 overexpression plasmid (pCMV-Sumo3). Red arrows indicate the time points undergoing UIRI, unilateral nephrectomy surgery (UNx) and sacrifice. **b** Quantitative data showing the serum creatinine levels in different groups. *** *P* < 0.001 versus sham controls; ^**†††**^*P* < 0.001 versus UIRI; ^###^*P* < 0.001 versus UIRI + pHA-Wnt1 (*n* = 5). **c** and **d** Representative western blotting and quantitative data showing protein expression of active β-catenin and Sumo3-LKB1 in different groups. Numbers (1–2) indicate individual animals in a given group. *P < 0.05, **P < 0.01 versus sham controls; ^**†**^*P* < 0.05, ^**††**^*P* < 0.01 versus UIRI; ^##^*P* < 0.01 versus UIRI + pHA-Wnt1 (*n* = 5). **e** Representative immunofluorescence micrographs showing renal β-catenin and Sumo3 expression in different groups. Frozen and paraffin sections were stained with antibodies against β-catenin or Sumo3. Arrows indicate positive staining; scale bar: 50 µm. **f–o** Representative western blotting and quantitative data showing protein expression of p-LKB1, LKB1, p-AMPKα/AMPKα, Fibronectin, α-SMA, p-ACC, CPT1A, PGC-1α and ACOX1 in different groups. Numbers (1–2) indicate individual animals in a given group. *P < 0.05, **P < 0.01, ***P < 0.001 versus sham controls; ^**†**^*P* < 0.05, ^**††**^*P* < 0.01, ^**†††**^*P* < 0.001 versus UIRI; ^#^*P* < 0.05, ^##^*P* < 0.01, ^###^*P* < 0.001 versus UIRI + pHA-Wnt1 (*n* = 5). **p** Representative immunofluorescence staining of Fibronectin (upper), CPT1A(middle) and Nile Red (bottom) in different groups. Frozen and paraffin sections were stained for Fibronectin or CPT1A expression and lipid accumulation (Nile Red). Arrows indicate positive staining; scale bar: 50 µm. **q** The electron microscopy image shows lipid droplets in different groups. Red stars indicate lipid droplets; scale bar: 2 µm. The data were analyzed by using one-way ANOVA.

UIRI mice performed intravenous injection of Wnt1 expression plasmid (pHA-Wnt1) or co-treated with Sumo3 expression plasmid (pCMV-Sumo3). Wnt1 is a typical ligand for triggering β-catenin activation, and also a secreted factor which could diffuse to neighboring cells. Hence, we injected mice with Wnt1 expressing plasmid to induce endogenous β-catenin activation. The experimental design is presented in Fig. 8a. The efficacy of Wnt1 or Sumo3 overexpression was confirmed by immunohistochemistry or immunofluorescence (Supplementary Fig. S1).

As shown in Fig. 8b, serum creatinine (Scr) level was significantly elevated in UIRI mice, but was further augmented by overexpression of Wnt1, however, co-treatment with Sumo3 inhibited it. The activation of β-catenin was induced, and Sumo3 was inhibited in UIRI mice, which was further exacerbated by ectopic Wnt1. However, overexpression of Sumo3 inhibited these effects (Fig. 8c–e). p-LKB1, LKB1, p-AMPKα, and AMPKα were further decreased by overexpression of Wnt1 in UIRI mice but were significantly reversed by overexpression of Sumo3 (Fig. 8f–i). Furthermore, as shown in Fig. 8f and j, k, Fibronectin and α-SMA were upregulated in UIRI mice, and further exacerbated by overexpression of Wnt1, but this was largely blocked by co-treatment with Sumo3. We then assessed the expression of metabolism-related proteins and found overexpression of Wnt1 further exacerbated the loss of p-ACC, CPT1A, PGC-1α, and ACOX1, but these effects were abolished by ectopic expression of Sumo3 (Fig. 8f, l–p). Similar results were observed when Fibronectin and CPT1A were examined by immunofluorescence (Fig. 8p). Lipid deposition was assessed by Nile red staining. As shown in Fig. 8p, Sumo3 blocked Wnt1-aggravated lipid accumulation. Transmission electron microscopy (TEM) analysis further clearly presented that Wnt1 overexpression-triggered lipid droplets were largely blocked by Sumo3 (Fig. 8q). ATP production assay showed that Sumo3 promoted ATP generation (Supplementary Fig. S1). This further suggested that Sumo3-mediated LKB1 Sumoylation plays a key role in regulating fatty acid metabolism and alleviating renal fibrosis.

### Sumo3 negatively correlates with the progression of CKD

**Fig. 9:**
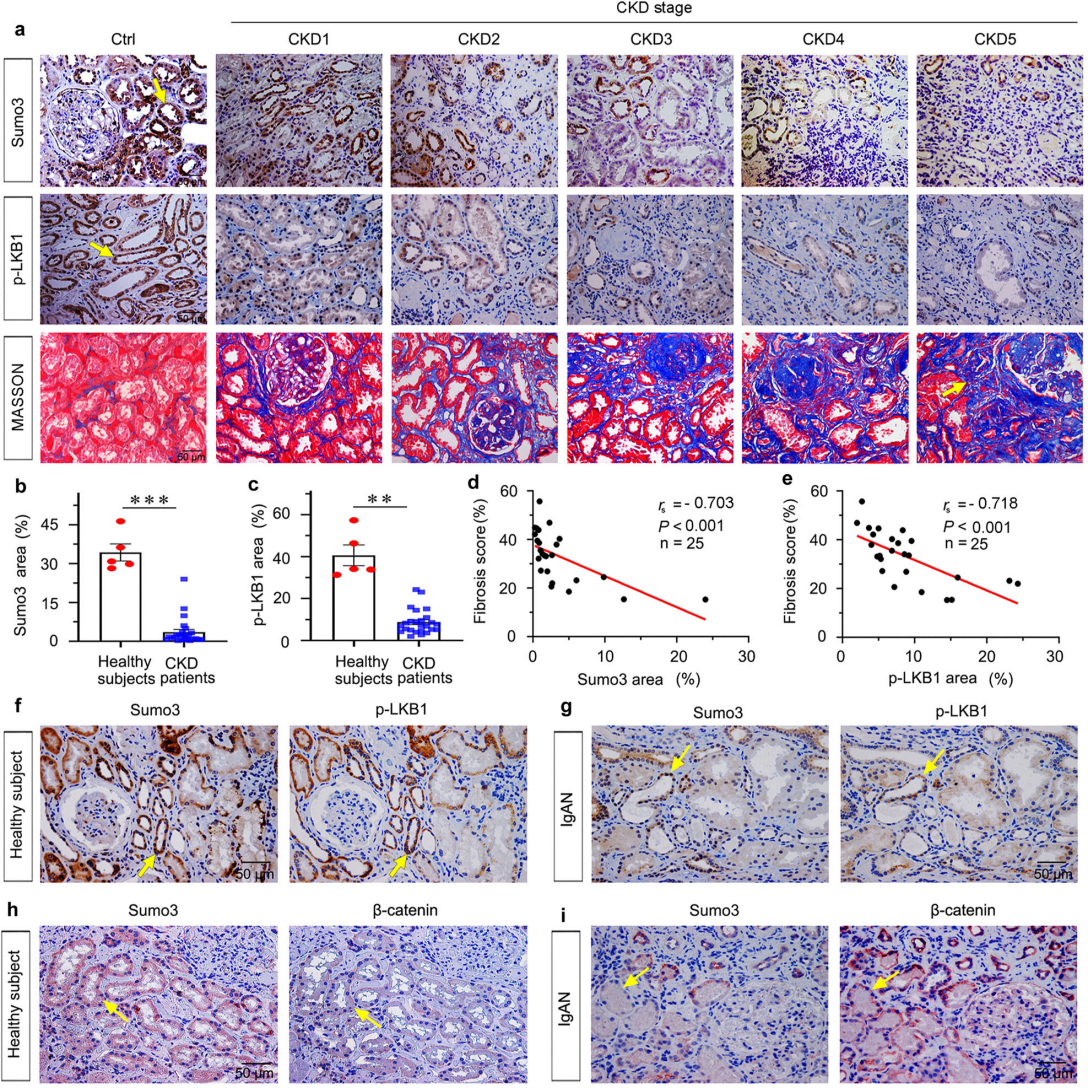
Sumo3 negatively correlates with the progression of CKD. **a** Representative micrographs showing renal expression of Sumo3 (upper) and p-LKB1 (middle) and Masson staining (bottom) to identify collagen deposition in healthy subjects and patients with IgA nephropathy from CKD1 to 5 stages. Paracancerous kidney tissue was used as healthy subjects. Arrows indicate positive staining (scale bar: 50 µm). **b** Quantitative analysis of immunohistochemical staining of Sumo3 in patients with CKD and healthy subjects. *n* = 5 (healthy subjects); *n* = 25 (CKD patients). ***P < 0.001 versus healthy subjects. **c** Quantitative analysis of immunohistochemical staining of p-LKB1 in patients with CKD and healthy subjects. *n* = 5 (healthy subjects); *n* = 25 (CKD patients). **P < 0.01 versus healthy subjects. **d** Linear regression showing the Spearman correlation coefficient (*r*_*s*_) and *P* value between Sumo3 and fibrosis score from CKD patients, respectively. *n* = 25. **e** Linear regression showing the Spearman correlation coefficient (*r*_s_) and *P* value between p-LKB1 and fibrosis score from CKD patients, respectively. *n* = 25. **f**, **g** Representative micrographs showing simultaneous staining of Sumo3 and p-LKB1 in kidney sections from healthy subjects or from IgA nephropathy patient. Sequential paraffin-embedded sections were subjected to stain with antibodies against Sumo3 or p-LKB1. Arrow indicates positive staining; scale bar: 50 µm. **h**, **i** Representative micrographs showing interlaced staining of Sumo3 and β-catenin in kidney sections from healthy subjects or from IgA nephropathy patients. Sequential paraffin-embedded sections were stained with antibodies against Sumo3 or β-catenin. Arrows indicate positive staining; scale bar: 50 µm. The data were analyzed by using Student’s *t*-test or Spearman correlation analysis.

We then adopted kidney biopsies from patients with IgA nephropathy (IgAN) at CKD1–5 stages. Sumo3, p-LKB1 expression and Masson’s trichrome (Masson) staining were assessed. Both Sumo3 and p-LKB1 proteins were mainly expressed in tubules in normal kidneys but also expressed in interstitium and glomeruli to a lesser extent. As shown, compared to the strongly positive signal in normal human kidneys, Sumo3 or p-LKB1 was decreased in CKD (Fig. 9a–c). With the progression of CKD, Sumo3 and p-LKB1 decreased gradually. There was a negative correlation between Sumo3 or p-LKB1 expression and kidney fibrosis (Fig. 9d, e). The staining on sequential sections further claimed, in healthy controls, a simultaneous expression of Sumo3 and p-LKB1 in many areas of renal tubules (Fig. 9f). But both Sumo3 and p-LKB1 showed the decreasing trend in same tubules in IgAN kidney (Fig. 9g). Furthermore, in healthy controls, Sumo3-positive tubules showed no expression of β-catenin (Fig. 9h). While in IgAN kidney, Sumo3 was lost in tubules with an increased expression of β-catenin (Fig. 9i). These results further suggest the negative regulation between Sumo3 and β-catenin, and Sumoylated LKB1 correlated with the progression of CKD.

### Fatty acid metabolism disorders play a central role in renal fibrosis and are closely related to β-catenin

**Fig. 10:**
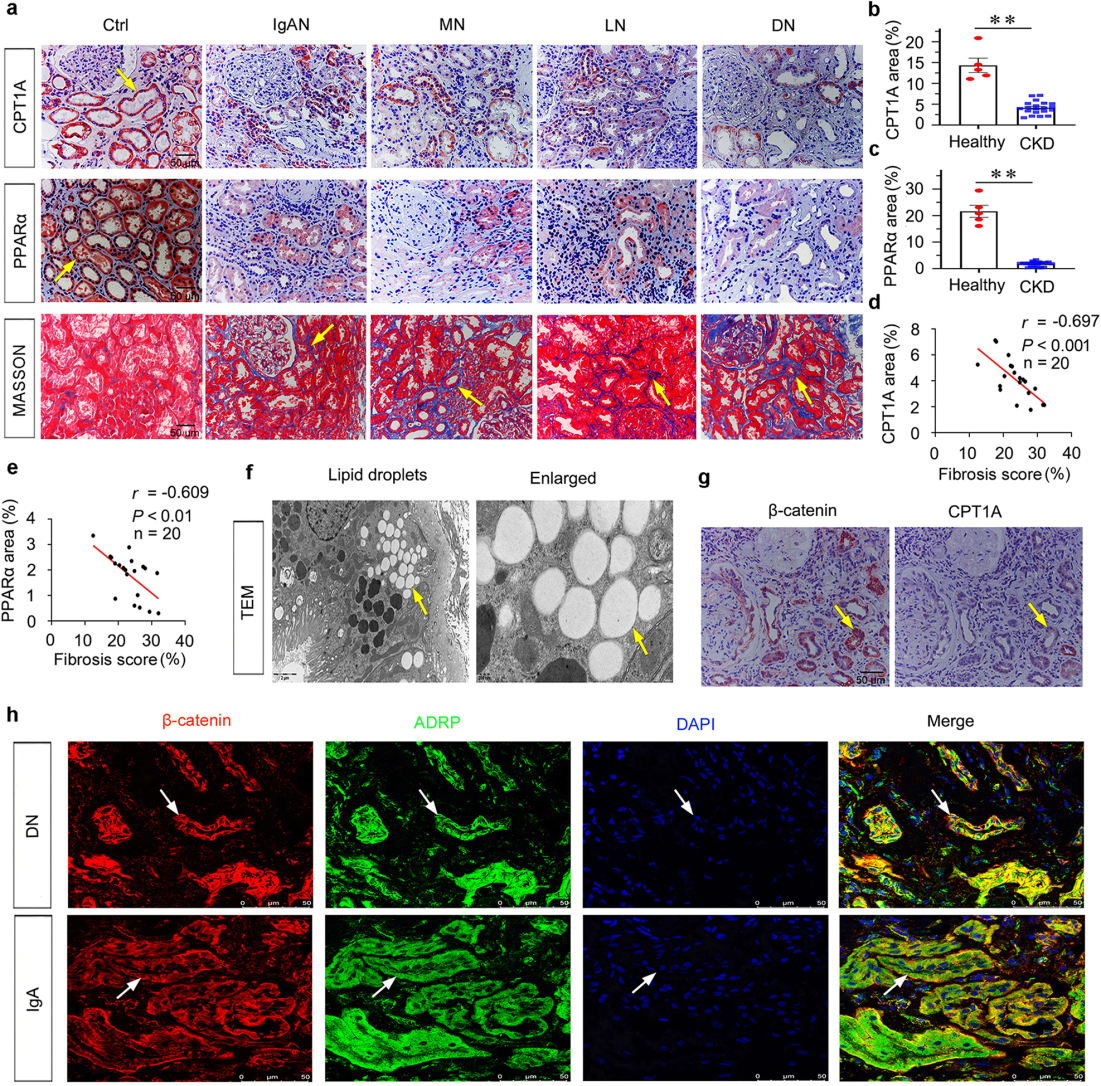
Fatty acid metabolism disorders play a central role in renal fibrosis and are closely related to β-catenin. **a** Representative micrographs showing renal expression of CPT1A (upper), PPARα (middle), as well as Masson staining (bottom) in healthy subjects and different patients with CKD. Human renal biopsy sections were obtained from healthy subjects (paracancerous tissue) and patients with IgA nephropathy (IgAN), membranous nephropathy (MN), lupus nephritis (LN), and diabetic nephropathy (DN). Arrows indicate positive staining (scale bar: 50 µm). **b** Quantitative analysis of immunohistochemical staining of CPT1A in patients with CKD and healthy subjects. *n* = 5 (healthy subjects); *n* = 20 (CKD patients). **P < 0.01 versus healthy subjects. **c** Quantitative analysis of immunohistochemical staining of PPARα in patients with CKD and healthy subjects. *n* = 5 (healthy subjects); *n* = 20 (CKD patients). **P < 0.01 versus healthy subjects. **d** Linear regression showing the Pearson correlation coefficient (*r*) and *P* value between CPT1A and fibrosis score from CKD patients, respectively. *n* = 20. **e** Linear regression showing the Pearson correlation coefficient (*r*) and *P* value between PPARα and fibrosis score from CKD patients, respectively. *n* = 20. **f** Representative transmission electron microscopy (TEM) images showing the lipid droplets in renal tubular epithelial cells in a patient with diabetic nephropathy. Arrows indicate positive staining; scale bar: 2 µm. **g** Representative micrographs showing the staggered localization of CPT1A and β-catenin in a patient with IgA nephropathy. Sequential kidney paraffin sections were stained with antibodies against β-catenin or CPT1A. Arrows indicate positive staining; scale bar: 50 µm. **h** Representative micrographs showing co-localization of β-catenin and ADRP in patients with IgAN and diabetic nephropathy (DN). Kidney frozen sections were stained for β-catenin (red) and ADRP (green) by immunofluorescence. Arrows indicate positive staining; scale bar: 50 µm. The data were analyzed by using Student’s t-test or Pearson correlation analysis.

In order to determine the critical role of β-catenin in modulating fatty acid metabolism, we examined the expression of CPT1A and PPARα, markers for fatty acid metabolism, in various clinical nephropathies. As shown in Fig. 10a–c, both CPT1A and PPARα proteins were predominantly localized in tubules, but less expressed in the interstitium and glomeruli in normal kidneys. Compared to the strongly positive signal in normal kidneys, CPT1A and PPARα were decreased in kidney biopsies from patients with CKD, including IgAN, membranous nephropathy (MN), lupus nephritis (LN) and diabetic nephropathy (DN).

We further assessed fibrotic lesions and found that there was a negative correlation between CPT1A or PPARα expression and renal fibrosis (Fig. 10a, d and e). Lipid droplets, in the kidney of CKD patients, were observed by electron microscopy (Fig. 10f). We further identified β-catenin-positive tubules with a loss of CPT1A (Fig. 10g). Furthermore, β-catenin upregulation was accompanied by an increase in ADRP, a lipid droplets marker, in various CKD patients’ kidneys (Fig. 10h). These results further identified β-catenin negatively controls FAO metabolism to contribute renal fibrosis.

## Discussion

Kidney fibrosis is a common pathological process in all types of CKD, affecting more than 10% of the population in the world [26, 27]. Of note, there are no effective strategies to cure renal fibrosis except renal replacement therapies such as dialysis or transplantation. However, the high costs of these therapies lead to huge economic burdens on healthcare [26–29]. Hence, finding a new therapeutic approach is critical for nephrologists. The best solution for this is to elucidate the pathogenesis of renal fibrosis, which is still a huge mystery [26, 30].

Renal tubular epithelial cells, which constitute the vast majority of kidney parenchyma, undertake a variety of physiological functions such as reabsorption of glucose, ions, proteins, and secretion of small molecular substances. Thereby, they possess a huge energy demand. Compared to glucose, renal tubular cells, especially proximal tubular cells (PTCs), are prone to metabolize fatty acids for their high efficiency in ATP production to meet high energy consumption [7, 11, 31]. Defects of fatty acid oxidation in renal tubular epithelial cells strongly promote lipotoxicity and renal tubulointerstitial fibrosis [7, 31]. The excessive free fatty acid could not only lead to the deposition of triacylglycerols in non-adipose tissue, but can also cause tissue or organ injury by triggering a series of damaging signals, such as the production of reactive oxygen species, apoptosis, pro-fibrogenesis, and pro-inflammation [7, 26, 32–36]. However, the underlying mechanisms of fatty acid metabolism disorders in the kidney remain elusive.

In the present study, we found that β-catenin, a detrimental signal for renal fibrosis, negatively regulates fatty acid metabolism in the kidney, which stimulates lipid accumulation. We further found that β-catenin regulates Sumoylated LKB1 by inhibiting Sumo3. The relationship between β-catenin and LKB1 Sumoylation was also identified in CKD patients, further suggesting the critical role of this signaling pathway. Therefore, we found a new mechanism of renal fibrosis that is highly involved in energy metabolism dysfunction in the kidney.

Interestingly, several studies have reported Sumo proteins exhibit rapid upregulation, followed by a subsequent return to normal levels in AKI models, reflecting an early response of tubular cell impairment [25]. In this work, we found a profound downregulation of Sumo proteins in both CKD models of UIRI and UUO mice, which may contribute to renal fibrosis. However, the key hypo-Sumoylated proteins in CKD should be identified. Although Sumoylation has been implicated in kidney fibrosis through regulating the TGF‐β and HIF‐1α pathway [37], we found LKB1 Sumoylation was strongly inhibited in this study. As the master controller for energy metabolism, LKB1 has been found to be fundamental for tubular cell energy control and cellular homeostasis [38]. LKB1 serves as an upstream kinase of AMPK, which is tightly regulated by post-translational modifications. For example, LKB1 can be phosphorylated by kinases like PKA, PKC, and ATM, specifically at Ser428, which promotes its binding and activation to AMPK [39, 40]. On the other hand, the Sumoylation of LKB1 is also crucial for its ability to enhance AMPK activity, thereby promoting cell energy metabolism [18]. Notably, Sumoylation is crucial to protein stability through Sumo molecules binding to targeted proteins. In this study, we found Sumo(s)-bond LKB1 protein was highly decreased, and importantly, β-catenin intimately regulates it.

β-catenin is the downstream effector of canonical Wnt signaling and is a versatile player in kidney injury and repair [41]. In the osteoblasts, Wnt-Lrp5 signaling, by way of activating β-catenin, regulates fatty acid β-oxidation [42]. The role of β-catenin in the regulation of fatty acid metabolism in the kidney has not been reported yet. In other organ studies, scientists find β-catenin inhibits LKB1 [22] to control cell fate [23]. However, it is still unknown whether β-catenin regulates fatty acid metabolism in the kidney through modulating LKB1-AMPK signaling.

In our study, we found that β-catenin-controlled Sumo modification of LKB1 plays a key role in energy metabolism in renal tubular cells during renal fibrosis. There are several lines of evidence supporting this finding. First of all, overexpression of Wnt/β-catenin signaling, in vivo and in vitro, inhibited LKB1 Sumoylation and disrupted fatty acid metabolism. While tubular cell-specific ablation of β-catenin preserved LKB1 Sumoylation, and restored fatty acid metabolism. Furthermore, large amounts of results demonstrated β-catenin modulates LKB1 Sumoylation by inhibiting Sumo3 expression. In addition, the supplementation of Sumo3 restored fatty acid metabolism and retarded renal fibrosis. Finally, in CKD patients, we found an intimate correlation among Sumo3, LKB1, and lipid depletion. These findings highlight a pivotal role of β-catenin in the setting of lipid metabolism through regulating Sumo modification of LKB1.

Another novel and interesting discovery, in this study is that only Sumo3 could completely restore LKB1 under the circumstance of β-catenin overexpression in renal tubular epithelial cells. Furthermore, Sumo3 greatly triggered the activation of p-AMPKα, and thus enhanced fatty acid metabolism. Reports showed Sumo modification of LKB1 on Lys178 mediates its localization and activation in tumor cells [18, 43]. Besides that, in renal tubular cells, we found Sumoylated residues of LKB1 are located at both Lys178 and Lys235 residues, suggesting the significant importance of Sumoylation of LKB1 in kidney cells. Additionally, we found fatty acid metabolism disorders are positively correlated with kidney fibrogenesis in CKD patients, consistent with previous studies [7, 11, 31]. This further implies that modulating LKB1 Sumoylation would be a more effective way to regulate energy metabolism during CKD, which is a potential approach to protect against lipotoxicity and renal fibrosis.

There are some limitations in this study. Ksp(Cadherin-16)-cre mice have some shortcomings, which mostly show gene ablation in collecting ducts, loops of Henle and distal tubules, and also exhibit gene deletion in proximal tubules to some extent [44]. From our studies, we can see under the control of Ksp-cre, β-catenin was deleted in collecting ducts and distal tubules and also was ablated in proximal tubules to some extent. Although proximal tubules mainly rely on FAO to supply energy, the TAL and cortical collecting ducts also depend on FAO to meet their energy demand [45]. Hence, we thought Ksp-cre mice were suitable for our study. However, we admit that using a proximal tubule-specific mouse model would be better, and this will be considered in future studies. Moreover, considering the adjacent expression of Cadherin-16 and β-catenin [46], it would be better to use Ksp-cre mice as wild-type controls to exclude these confounding factors in future studies.

Another limitation is that we did not adopt frozen kidney sections from CKD patients to assess the co-localization of Sumo3 and LKB1. However, due to the difficulties in acquiring and preserving the frozen sections from patients, we adopted sequential paraffin-embedded sections for detecting the co-expression of LKB1 and Sumo3 in the same tubules, which is a good substitute for testing two proteins in one frozen section. In the future, more frozen sections should be used to demonstrate the co-localization of Sumo and LKB1 with an image-overlapping study.

In summary, we found a new mechanism of fatty acid metabolism dysfunction in renal fibrosis. Furthermore, we found that β-catenin controls lipid metabolism through regulating LKB1 Sumoylation. Sumo3, as a downstream target of β-catenin, could be a potential therapeutic target in fighting against renal fibrosis.

## Methods and materials

### Human kidney biopsy samples

All human kidney biopsy specimens were collected from patients with CKD at the Fifth Affiliated Hospital of Sun Yat-sen University following the provision of written consent. The demographic and clinical data are presented in Supplementary Table S1. Human specimens were obtained from kidney biopsies with pathological diagnosis. All studies involving human kidney biopsy samples were approved by the Ethics Committee on Human Subjects of the fifth affiliated hospital of Sun Yat-Sen University (2023-K136-1).

### Animal models

Male C57BL/6 mice aged 6–8 weeks were purchased from the Experimental Animal Center of Southern Medical University (Guangzhou, China). Tubular epithelial cells-specific β-catenin conditional knockout mice (Ksp-β-catenin^−/−^) were purchased from Cyagen Biosciences (Guangzhou, China), which were created by mating β-catenin floxed mice with Ksp-cre transgenic mice. The stock number was CKOCMP-12387-Ctnnb1-B6N-VA. The genotyping of tail DNA samples was performed by PCR protocol according to the manufacturer’s instructions as previously reported [36]. For the UUO model, after a midline abdominal incision, the left ureter was dissociated from the surrounding tissues and mice received permanent ligation at the opening of the renal pelvis and the upper third of the ureter. For the UIRI model, mice were randomly grouped before surgery. The left renal pedicle was clipped with a noninvasive arterial clip for 30 min, and the body temperature was maintained at 37.8 °C. On the 10th day of surgery, the right kidney was excised, and the mice were sacrificed on the 11th day of surgery. In vivo, expression of Sumo3 or Wnt1 in mice was performed using a hydrodynamic-based gene delivery approach. The detailed experimental designs are shown in Fig. 8a. The animal experiments were authorized by the Animal Ethics Committee of Southern Medical University, Guangzhou, China (NFYY-20230704-001).

### Transmission electron microscopy

To assess kidney tubular epithelial lipid accumulation, the handling of electron microscopic samples was performed according to procedures established at the KingMed Center. In brief, the kidney cortex was collected and fixed in 1.25% glutaraldehyde/0.1 M phosphate buffer, then ultrathin sections (60 nm) were prepared, and slides were examined under an electron microscope (JEM-1400 PLUS, Tokyo, Japan).

### Determination of serum creatinine

Serum creatinine (Scr) was determined by an automatic chemistry analyzer. The Scr was expressed as milligrams per deciliter.

### Nile red staining

Nile red staining was according to the manufacturer’s instructions. In brief, HKC-8 cells cultured on coverslips were fixed with 2% formaldehyde for 10 min at room temperature. Firstly, wash the slides two times with PBS, then wash the slides with PBS containing 0.1% Tween 20 for 5 min. Secondly, decant extra liquid on the slides. Finally, add PBS containing both Nile red (1 μg/ml) and DAPI (1 μg/ml) on the slides and incubate in the dark room for 10 min. All images were taken by confocal microscopy (Leica TCS SP2 AOBS, Leica Microsystems, Buffalo Grove, IL).

### Cell culture and treatment

Human proximal tubular epithelial cells (HKC-8) culture was performed according to procedures described previously [47]. HKC-8 cells were incubated with TGF-β (2 ng/ml) or transfected with stabilized β‐catenin expression vector (pDel‐β‐catenin), human Sumo1 expression plasmid (pCMV-Sumo1), human Sumo2 expression plasmid (pCMV-Sumo2), as well as human Sumo3 expression plasmid (pCMV-Sumo3) using Lipofectiamine 2000. Some cells were transfected with pDel‐β‐catenin alone or with pCMV-Sumo3, or co-treated with etomoxir (40 μM, HY-50202; MCE) for 18 h, and then all added with Sodium oleate (200 μM, HY-N1446B; MCE) for 24 h as previously reported [48, 49]. Whole-cell lysates were prepared and subjected to real‐time PCR or Western blotting analyses. Some cells were also tested by immunofluorescence staining, Nile Red staining, chromatin immunoprecipitation, co-immunoprecipitation or luciferase analyses.

### Western blotting analysis

Protein expression was analyzed by western blotting analysis as described previously [37]. The following primary antibodies were used: anti-β-catenin (BD Biosciences, Cat. #610154, 1:2000), anti-Sumo1 (Cell Signaling Technology, Cat. #4930s, 1:1000), anti-Sumo2/3 (Cell Signaling Technology, Cat. #4971s, 1:1000), anti-Sumo3 (Abcam, Cat. #ab203570, 1:1000), anti-LKB1 (Santa Cruz Biotechnology, Cat. #sc-32245, 1:1000), anti-p-LKB1 (Cell Signaling Technology, Cat. #3482s, 1:1000), anti-AMPKα (Cell Signaling Technology, Cat. #2532s, 1:1000), anti-p-AMPKα (Cell Signaling Technology, Cat. #2535s, 1:1000), anti-α-tubulin (Beijing Ray Antibody Biotech, Cat. #RM2007, 1:5000), anti-fibronectin (Sigma, Cat. #F3648, 1:50,000), anti-α-SMA (Abcam, Cat. #ab5648, 1:1000), anti-p-ACC (Cell Signaling, Cat. #3661s, 1:1000), anti-CPT1A (Abcam, Cat. #ab5648, 1:1000), anti-PGC-1α (Abcam, Cat. #ab54481, 1:1000), anti-ACOX1 (ABclonal, Cat. #A8091; 1:1000), anti-PPARα (ABclonal, Cat. #A6697, 1:1000), anti-TOMM20 (Abcam, Cat. #ab186735, 1:1000), anti-active-β-catenin (Cell Signaling Technology, Cat. #19807s, 1:1000), anti-β-actin (Ray Antibody Biotech, Cat. #RM001, 1:5000), and anti-Flag (Proteintech, Cat. #20543-1-AP, 1:1000).

### Transcriptomic analysis

The data of transcriptome sequencing have been used in our previous study [50] and is available at NCBI with accession number GSE193282. In brief, total RNA was isolated according to the manufacturer’s instructions. The primary experimental procedures for transcriptome sequencing analysis include RNA quantification and qualification, library preparation for transcriptome sequencing, clustering and sequencing, and data analysis. featureCounts v1.5.0-p3 was used to count the reads numbers mapped to each gene. Then, the FPKM of each gene was calculated based on the length of the gene and the read count mapped to this gene. Differential expression analysis of two conditions was performed using the DESeq2 R package (1.20.0). Genes with an adjusted *P* < 0.05 found by DESeq2 were assigned as differentially expressed. GO enrichment analysis of differentially expressed genes was implemented by the clusterProfiler R package, in which gene length bias was corrected. We also used clusterProfiler R package to test the statistical enrichment of differential expression genes in KEGG pathways. GO terms with corrected *P* < 0.05 were considered significantly enriched by differentially expressed genes. The GSEA web interface with the Molecular Signatures Database “Fatty acid metabolism,” “PPARA pathway,” and “LKB1-AMPKα signaling” genesets were used to show a significant, consistent difference between the two biological states. We use the local version of the GSEA analysis tool http://www.broadinstitute.org/gsea/index.jsp.

### Immunofluorescence staining

Frozen sections of mouse or human kidneys were fixed with 4% paraformaldehyde for 15 min at room temperature. HKC-8 cells cultured on coverslips were fixed with cold methanol:acetone (1:1) for 15 min at room temperature. After permeabilizing with 0.2% Triton X-100 for 10 min, then blocked slides with 10% normal donkey serum for 1 h. Slides were incubated with primary antibodies against anti-β-catenin (BD Biosciences, Cat. 610154, 1:150), anti-Sumo3 (Abcam, Cat.ab203570, 1:50), anti-ADRP (Abcam, Cat. ab52356, 1:50), anti-fibronectin (Sigma, Cat. F3648; 1:100), anti-p-AMPKα (Cell Signaling Technology, Cat. #2535s, 1:50) and anti-CPT1A (Abcam, Cat. ab128568, 1:50), anti-Lotus Tetragonolobus Lectin (LTL) (VECTOR Laboratories, Cat. FL-1321,1:400), anti-Peanut Agglutinin (PNA) (VECTOR Laboratories, Cat. FL-107, 1:400), anti-Dolichos Biflorus Agglutinin (DBA) (VECTOR Laboratories, Cat. #FL1031, 1:400), and anti-E-cadherin (Cell Signaling Technology, Cat. #3195s, 1:1000). After washing with TBS-T, slides were incubated with Cy2 or Cy3-conjugated donkey anti-mouse or anti-rabbit IgG (Jackson Immuno-Research Laboratories, West Grove, PA, USA). Nuclei were stained with DAPI (Beyotime, Cat. #C1006) according to the manufacturer’s instructions. All images were taken by confocal microscopy (Leica TCS SP2 AOBS, Leica Microsystems, Buffalo Grove, IL, USA).

### Histology and immunohistochemical staining

Paraffin-embedded human kidney (2.5-μm thick) and mouse kidney (4-μm thick) sections were prepared by a routine procedure. The sections were stained with Masson staining according to the manufacturer’s protocol. Immunohistochemical staining was performed as described previously. The following antibodies were used: anti-CPT1A (Abcam, Cat. ab128568, 1:50), anti-PPARα (ABclonal, Cat. A6697, 1:50), anti-β-catenin (Abcam, Cat. ab189524, 1:50), anti-β-catenin (BD Biosciences, Cat. 610154, 1:150), anti-PGC-1α (Abcam, Cat.ab54481, 1:50), anti-Sumo1 (Cell Signaling Technology, Cat. #4930s, 1:50), anti-Sumo2/3 (Cell Signaling Technology, Cat. #4971s, 1:50), anti-Sumo3 (Abcam, Cat.ab203570, 1:1000) anti-p-LKB1 (Cell Signaling Technology, Cat. #3482s, 1:50). Images were taken by an Olympus DP80 microscope with EMCCD camera.

### Real-time qRT-PCR

Total RNA was acquired using TRIzol RNA isolation system (Life Technologies, Grand Island, NY) according to the manufacturer's instructions. The first strand of complementary DNA was synthesized by using a Promega reverse transcription (Promega, Madison, WI), and Real-time PCR was performed on an ABI prism 7000 Sequence Detection System (Applied Biosystems, Foster City, CA). The sequences of the primer pairs used in Real-time qRT-PCR were shown in Supplementary Table S2. The mRNA levels of various genes were calculated after normalizing by β-actin.

### Immunoprecipitation (IP)

The interaction of LKB1 and Sumos was determined by immunoprecipitation as previously described [51]. HKC-8 cells were transfected with a stabilized β‐catenin expression vector (pDel‐β‐catenin) or pcDNA3 using Lipofectamine 2000 for 24 h. Cell lysates or kidney homogenates were immunoprecipitated overnight at 4 °C with an anti-LKB1 antibody (Santa Cruz Biotechnology, sc-32245) and protein A/G plus agarose (sc-2003; Santa Cruz Biotechnology). The precipitated complexes were washed three times with lysis buffer and boiled for 5 min in SDS sample buffer, followed by immunoblotting with anti-Sumo1 (Cell Signaling Technology, Cat.#4930s), anti-Sumo2/3 (Cell Signaling Technology, Cat.#4971s) or anti-Sumo3 (Abcam, Cat.ab203570, 1:1000), respectively.

### Chromatin immunoprecipitation (ChIP)

HKC-8 cells were transfected with β-catenin overexpression plasmid (pDel-β-catenin) or pcDNA3 for 6 h and continued to be incubated for an additional 24 h in a complete medium. Then, the cells were harvested and fixed with 4% formaldehyde for 10 min at room temperature for protein–DNA crosslinking. Cell lysates were obtained, and the SimpleChIP® Plus (Magnetic Bead) Kit (Cell Signaling, Cat. 9005) was used to perform the ChIP assay. The antibodies against TCF4 (Cell Signaling Technology, Cat. #2565s), H3, as positive control, and IgG, as negative control, were added, respectively, and incubated overnight at 4 °C, followed by incubation with magnetic beads for 1 h. After washing out the precipitate, purified DNA was used as a template for PCR. The sequences of human Sumo3 primers were as follows: Binding site 2, forward 5′-cctttttgcatctttgctgtc-3′ and reverse 5′-aattacgccagttgctgtgtc-3′; Binding site 3, forward 5′-ccagtgcctcagtttcttcac-3′ and reverse 5′-gccagcaccctagacgatt-3′.

### Quantification of renal immunohistochemically positive areas and fibrosis

An Olympus DP80 microscope with an EMCCD camera was used to observe the glass slides stained with CPT1A, PPARα, Sumo3, p-LKB1 and Masson stain under the microscope, and 2448 × 1920 pixel resolution images captured at a high magnification (×400) field from a randomly selected field were captured. Each part contains 10 fields, and the Image Pro Plus was used to measure the area of the lesion area and the total area of each part. And then, the lesion area divided by the total area was the percentage of the immunohistochemically positive area or fibrosis.

### Determination of ATP

Cells or tissues were harvested and fully lysed according to the manufacturer’s instructions. Then, the lysed cells or tissues were centrifuged at 4 °C, 12,000×*g* for 5 min to collect the supernatant. Finally, ATP production was determined by an enhanced ATP assay kit (Beyotime Biotechnology, S0027). All values were normalized to the controls.

### Luciferase assay

The effect of β-catenin on TCF4-mediated gene transcription was detected using a luciferase reporter assay kit (E1910, Promega, Madison, WI, USA). Briefly, HKC-8 cells were seeded in a 12-well plate and transfected with pGL3-Sumo3 constructed with the complete human promoter sequence with or without pDel-β-catenin, siRNA to TCF4 and pFlag-TCF4, using Lipofectamine 2000 reagent. Renilla luciferase was used as an internal control reporter. The luciferase assay was conducted according to the manufacturer’s protocols. Relative luciferase activity (arbitrary units) was calculated as fold induction over the controls after normalizing the transfection efficiency.

### Isolation of primary renal tubular epithelial cells and treatment

We isolated the primary renal tubular epithelial cells from male C57BL/6 following a routine protocol. The kidneys were minced, digested in collagenase IV and then filtrated with a 100-μm cell strainer. After centrifugation, the tubules were resuspended in DMEM/F12 with 10% FBS medium and were seeded in a six-well plate. After growing the cells for 72 h, the cells were co-transfected using IMVI DNA RNA Transfection Reagent (Invigentech^TM^, cat:IV1216150) with pDel-β-catenin and Sumo3 overexpression plasmid for 36 h. We then harvested the cells for western blotting and Immunofluorescence staining analysis.

### Sumo protein purification

HKC8 cells were transfected with the His_6_-Sumo1,2,3 plasmid and other respective plasmids as described. After treatment, the total cells protein was lysed by sonication, and part of cell lysates was not incubated with magnetic beads and served as input, others were for His_6_-Sumoylated proteins. The His_6_-Sumoylated proteins were then purified using HisSep Ni-NTA MagBeads (20561ES08, Yeasen company, China) and 250 mM imidazole solution as elution buffer protein.

### Statistical analyses

All data were presented as mean ± SEM. Statistical analysis was carried out using SPSS 19.0 (SPSS Inc., Chicago, IL, USA). Comparisons were made using Student’s *t*-test for comparison of two groups, or one-way ANOVA followed by the least significant difference or Games–Howell procedure for comparing more than two groups. *P* < 0.05 was considered statistically significant. Bivariate correlation analysis was analyzed by using Pearson correlation analysis or Spearman correlation analysis.

## Supplementary information

Supplementary material

Supplementary Figure S1

Supplementary Figure S2

Supplementary Figure S3

Full unedited gels for Western blot

## Data Availability

All data used in this work are available from the corresponding author upon reasonable request. The transcriptomic data can be acquired in Gene Expression Omnibus (GEO) datasets (https://www.ncbi.nlm.nih.gov/geo/) with accession number GSE193282.
