# Supplementary material for: β-catenin-inhibited Sumoylation modification of LKB1 and fatty acid metabolism is critical in renal fibrosis
Source: Cell Death Dis. 2024 Oct 22;15(10):769. doi: 10.1038/s41419-024-07154-y (PMC11496881; doi:10.1038/s41419-024-07154-y)
Supplement: Supplementary file 1 — Supplementary material [file 41419_2024_7154_MOESM1_ESM.docx]

**Supplementary material for**

**β-catenin-inhibited Sumoylation modification of LKB1 and fatty acid metabolism is critical in renal fibrosis**

Shuangqin Chen^#^, Jiemei Li^#^, Ye Liang^#^, Meijia Zhang^#^, Ziqi Qiu^#^, Sirui Liu^#^, HaoRan Wang, Ye Zhu, Shicong Song, Xiaotao Hou, Canzhen Liu, Qinyu Wu, Mingsheng Zhu, Weiwei Shen, Jinhua Miao, Fan Fan Hou, Youhua Liu, Cheng Wang*, Lili Zhou*

These authors contributed equally to this work.

This file includes:

Supplementary Table S1, S2

Supplementary Figure legends

*Corresponding author:

Dr. Lili Zhou, Division of Nephrology, Nanfang Hospital, 1838 North Guangzhou Ave, Guangzhou 510515, China, E-mail: [jinli730@smu.edu.cn;](mailto:jinli730@smu.edu.cn;)

Dr. Cheng Wang, Division of Nephrology, Department of medicine, The Fifth Affiliated Hospital Sun Yat-Sen University, Zhuhai, Guangdong 519000, China, E-mail: wangch2@mail.sysu.edu.cn.

**Supplementary Tables**

Table S1. Demographic and clinical data of human kidney biopsies samples.

|  | **No.** | **Gender*** | **Age** | **Pathological diagnosis ^#^** | **eGFR(ml/min/1.73m^2)** |
| --- | --- | --- | --- | --- | --- |
| Healthy Adults | 1 | M | 52 | No | 106 |
|  | 2 | M | 42 | No | 117 |
|  | 3 | F | 32 | No | 113 |
|  | 4 | M | 42 | No | 103 |
|  | 5 | M | 39 | No | 113 |
|  | 6 | F | 39 | No | 111 |
| Patients | 1 | F | 23 | IgAN | 113 |
|  | 2 | F | 44 | IgAN | 103 |
|  | 3 | F | 40 | IgAN | 120 |
|  | 4 | M | 65 | IgAN | 95 |
|  | 5 | M | 59 | IgAN | 93 |
|  | 6 | F | 45 | IgAN | 65 |
|  | 7 | M | 24 | IgAN | 67 |
|  | 8 | M | 43 | IgAN | 68 |
|  | 9 | F | 33 | IgAN | 74 |
|  | 10 | M | 72 | IgAN | 68 |
|  | 11 | M | 57 | IgAN | 40 |
|  | 12 | M | 54 | IgAN | 58 |
|  | 13 | M | 42 | IgAN | 57 |
|  | 14 | M | 64 | IgAN | 55 |
|  | 15 | M | 44 | IgAN | 51 |
|  | 16 | F | 46 | IgAN | 28 |
|  | 17 | M | 63 | IgAN | 23 |
|  | 18 | F | 34 | IgAN | 27 |
|  | 19 | F | 69 | IgAN | 20 |
|  | 20 | M | 48 | IgAN | 22 |
|  | 21 | F | 30 | IgAN | 5 |
|  | 22 | M | 42 | IgAN | 13 |
|  | 23 | M | 33 | IgAN | 14 |
|  | 24 | M | 45 | IgAN | 8 |
|  | 25 | F | 50 | IgAN | 13 |
|  | 26 | F | 46 | IgAN | 23.34 |
|  | 27 | M | 31 | IgAN | 64.43 |
|  | 28 | M | 64 | IgAN | 56.48 |
|  | 29 | M | 34 | IgAN | 84.66 |
|  | 30 | M | 65 | IgAN | 93.75 |
|  | 31 | F | 30 | LN | 121.59 |
|  | 32 | F | 24 | LN | 123.19 |
|  | 33 | F | 27 | LN | 141.9 |
|  | 34 | F | 33 | LN | 71.42 |
|  | 35 | M | 50 | MN | 116.71 |
|  | 36 | M | 46 | MN | 87.93 |
|  | 37 | F | 57 | MN | 101.84 |
|  | 38 | M | 69 | MN | 94.72 |
|  | 39 | M | 48 | MN | 104.56 |
|  | 40 | M | 63 | DN | 35.41 |
|  | 41 | M | 58 | DN | 56.51 |
|  | 42 | M | 61 | DN | 87.28 |
|  | 43 | M | 48 | DN | 6.57 |
|  | 44 | M | 51 | DN | 34.42 |
|  | 45 | F | 27 | LN | 56.73 |

*M, male; F, female. ^#^ MN, Membranous nephropathy; IgAN, IgA nephropathy; LN, Lupus nephritis; DN, Diabetes nephropathy; GFR: Glomerular filtration rate (ml/min/1.73m2).

Table S2. Primary primer sequences.

| **Primers** | | |
| --- | --- | --- |
|  |  |  |
| Human Sumo1 | F | TGTGGGGAAGGGAGAAGGATT |
|  | R | ATCCCCCAAGTCCTCAGTTGA |
| Human Sumo2 | F | GAAAAGCCCAAGGAAGGAGTCAA |
|  | R | TTTTCAGTAGACACCTCCCGTCT |
| Human Sumo3 | F | GGACACCATCGACGTGTTCC |
|  | R | CTCGAGTTTCCGCAGACACC |
| Mouse Ubc9 | F | TCATCCAAACGTGTATCCTTCTG |
|  | R | CTTGTGCTCGGACCCTTTTCT |
| Mouse SAE1 | F | CAGTATGACCGACAGATCCGC |
|  | R | GGAGATACCTGTTCGTGGTCC |
| Mouse SAE2 | F | CCACATCGACCTGATTGATCTG |
|  | R | GGCAACCTGAGCCTTTGATCT |
| Mouse RanBP2 | F | GCTGGCTGCATTGTGCTATC |
|  | R | GTGGGCCATCGTTTCCAGG |
| Mouse PIAS1 | F | GCGGACAGTGCGGAACTAAA |
|  | R | ATGCAGGGCTTTTGTAAGAAGT |
| Mouse PIAS2 | F | AGACTACTCAGTACCATTCCACC |
|  | R | CTCCAAGCTGCACTTCGTTAT |
| Mouse PIAS3 | F | GAAGGAGGCATCAGAGGTTTG |
|  | R | TAGACAGGAAATCACTGCCCA |
| Mouse PIAS4 | F | AGCTGTATGAGACTCGCTATGC |
|  | R | TGAAGAAGGGGAGCTTAACCAG |
| Mouse Pc2 | F | GGATCATTGCGTCGTACAGTG |
|  | R | TGCGTTATACACCGAGAAGTCTA |
| Mouse Sumo1 | F | CGCAAGACGTAGAGGAAGTCC |
|  | R | GTTTTGCCTCCTGGTCAGACA |
| Mouse Sumo2 | F | ATTGTGAACGGCAGGGTTTGT |
|  | R | CAACTGTGCAGGTGTGTCTGT |
| Mouse Sumo3 | F | GAGGCAGATTCGATTCCGGTTT |
|  | R | GTGTCCTCATCCTCCATCTCCA |
| Human β-actin | F | CTCACCATGGATGATGATATCGC |
|  | R | AGGAATCCTTCTGACCCATGC |
| Mouse β-actin | F | CAGCTGAGAGGGAAATCGTG |
|  | R | CGTTGCCAATAGTGATGACC |

**Supplementary Figure legends**

***Supplementary Figure S1. Sumoylation was inhibited in UIRI model***

**(a)** Representative electron microscopy image showing lipid droplets in UIRI mice. Arrows indicate lipid droplets; scale bar: 2 µm. **(b)** Quantitative data showing mRNA levels of Sumo1, Sumo2, and Sumo3 in different groups. ****P* < 0.001 versus sham controls (n = 5). **(c)** Quantitative data showing mRNA levels of SAE1, SAE2, Ubc9, PLAS1, PLAS2, PLAS3, PLAS4, Ranbp2 and Pc2 in different groups. ***P* < 0.01, ****P* < 0.001 versus sham controls (n = 5). **(d)** Quantitative data showing renal expression of p-AMPKα/AMPKα, p-ACC, CPT1A, ACOX1, PGC-1α and Fibronectin in UIRI and sham mice. **P* < 0.05, ***P* < 0.01, ****P* < 0.001 versus sham controls (n = 5). **(e)** Quantitative data showing ATP levels in different groups. ****P* < 0.001 versus sham controls; **^††^***P* < 0.01 versus UIRI; ^##^*P* < 0.01versus UIRI + pHA-Wnt1 (n = 5). **(f)** Representative immunofluorescence and immunohistochemical staining of Sumo3 tagged with EGFP (upper) and Wnt1 tagged with HA (bottom). Kidney frozen sections were stained with nuclear dye (DAPI) and observed for Sumo3 tag expression under a fluorescence microscope. Paraffin sections of kidneys were stained for Wnt1 tag (HA). Arrows indicate positive staining; For Sumo3 staining, scale bar: 100 µm. For Wnt1 staining, scale bar: 50 µm. The data were analyzed by using Student’s t-test or one-way ANOVA.

***Supplementary Figure S2.*** ***Sumo3 plays a decisive role in LKB1/AMPK signaling axis***

**(a-b)** Western blotting and quantitative data showing protein expression of active β-catenin in different groups. HKC-8 cells were transfected with pDel-β-catenin plasmid (or pcDNA3) for 24 h. ****P* < 0.001 versus pcDNA3. Numbers (1 to 4) indicate individual treatments in a given group. (n = 4) **(c)** Quantitative data showing the levels of ATP in different groups. HKC-8 cells were transfected with pDel-β-catenin and pCMV-Sumo3 (or pcDNA3) for 24 h. ****P* < 0.001 versus pcDNA3; **^††^***P* < 0.01 versus pDel-β-catenin (n = 3). **(d)** Representative micrographs showing fluorescence field (EGFP tags) for Sumo1, Sumo2, and Sumo3 and the bright field in different groups. Arrow indicates positive staining. Scale bar: 50 µm. **(e-j)** Representative western blotting and quantitative data showing protein expression of LKB1, p-AMPKα/AMPKα, p-ACC, CPT1A and Fibronectin in different groups. HKC-8 cells were stimulated with TGF-β1 recombinant protein and co-treated with pCMV-Sumo3. **P* < 0.05 versus pcDNA3; **^†^***P* < 0.05, **^††^***P* < 0.01 versus TGF-β1 (n = 3). The data were analyzed by using Student’s t-test or one-way ANOVA.

***Supplementary Figure S3. Knockout of β-catenin increased LKB1 Sumoylation in UUO model***

**(a)** Representative micrographs and quantitative analysis showing fibrotic area evaluated by Sirius red staining in different groups. Paraffin sections of mouse kidneys were performed to Sirius red staining. Scale bar: 50 µm. At least 10 randomly selected fields were evaluated under 400× magnification and results were averaged for each animal. ****P* < 0.001 versus sham controls; **^††^***P* < 0.01 versus WT UUO mice (n = 5). **(b)** Quantitative data showing the level of ATP in different groups. ****P* < 0.001 versus sham controls; **^†††^***P* < 0.001 versus WT UUO mice (n = 5). **(c)** Quantitative real-time PCR analysis showing mRNA levels of SAE1, SAE2, Ubc9, PLAS1, PLAS2, PLAS3, PLAS4, Ranbp2 and Pc2 in different groups. **P* < 0.05, ***P* < 0.01, ****P* < 0.001 versus sham controls; **^†^***P* < 0.05, **^††^***P* < 0.01, **^†††^***P* < 0.001 versus WT UUO mice (n = 5). **(d)** Representative co-staining of β-catenin and different segmental markers of tubules showing the KSP-cre-mediated conditional knockout of β-catenin leading to the ablation of β-catenin in distal tubules, connecting tubules, as well as the most parts of proximal tubules. Kidney sections were co-stained for β-catenin (Red) and various segment-specific tubular markers (green), respectively. The markers were used as follows: proximal tubule, lotus tetragonolobus, lectin (LTL); distal tubule, peanut agglutinin (PNA); and collecting duct, dolichos biflorus agglutinin (DBA). Scale bar, 25 µm. **(e)** Quantitative data showing quantification of β-catenin staining IOD (Integrated Optical Density) in tubule with positive staining for tubular markers, including LTL, PNA and DBA. At least 50 randomly selected tubules were evaluated and results were averaged for each animal. ****P* < 0.001 versus WT UUO mice (n = 5). **(f)** Representative immunohistochemical staining of β-catenin in different groups. Paraffin sections were stained with antibody against β-catenin. The upper panel provides a panoramic view of the complete field captured, and the lower panel shows a magnified image of the local area. Arrow indicates positive staining; scale bar: 50 µm. The data were analyzed by one-way ANOVA.
