# Supplementary figures and images for: β-catenin-inhibited Sumoylation modification of LKB1 and fatty acid metabolism is critical in renal fibrosis
Source: Cell Death Dis. 2024 Oct 22;15(10):769. doi: 10.1038/s41419-024-07154-y (PMC11496881; doi:10.1038/s41419-024-07154-y)

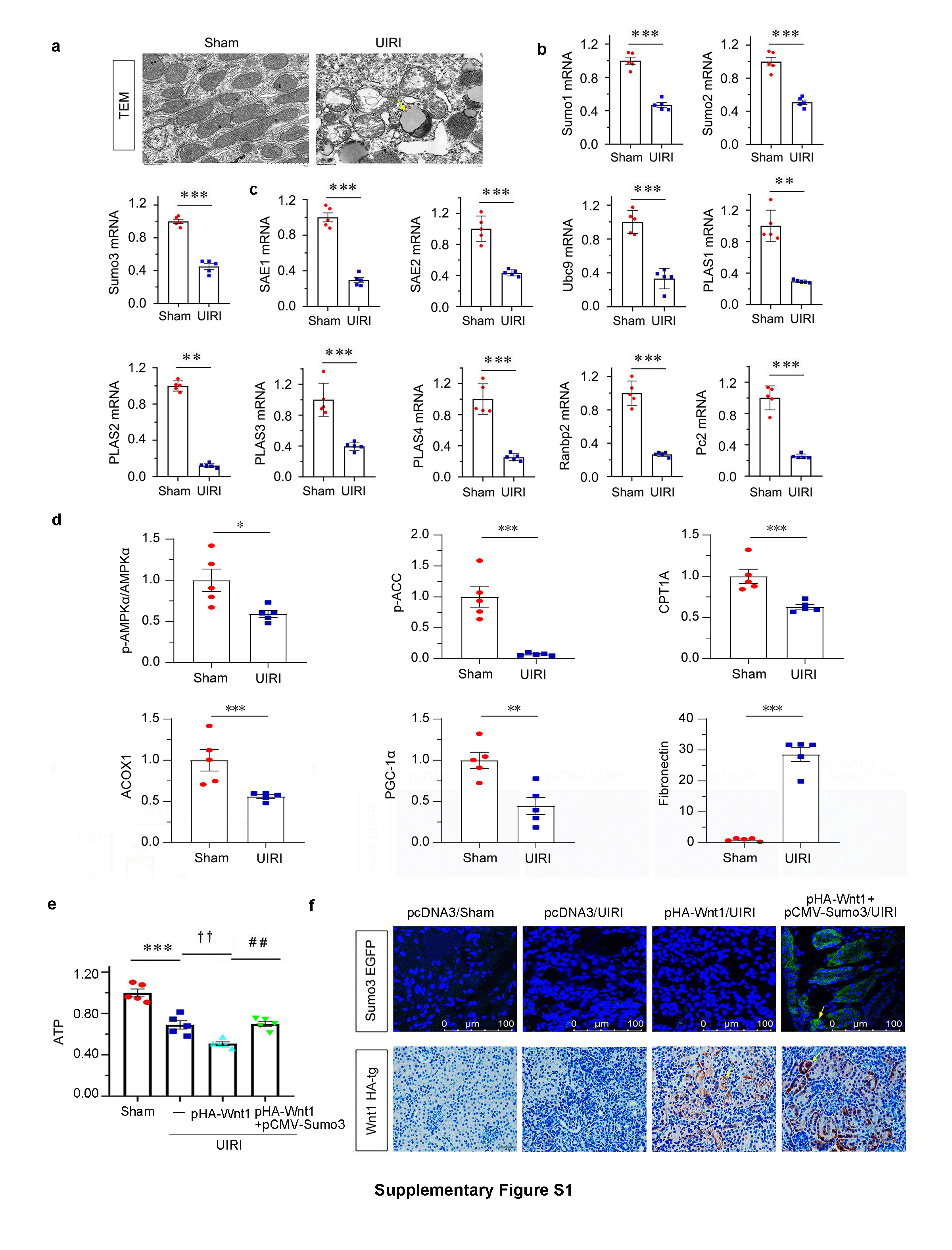

Supplement: Supplementary file 2 — Supplementary Figure S1 [file 41419_2024_7154_MOESM2_ESM.png]

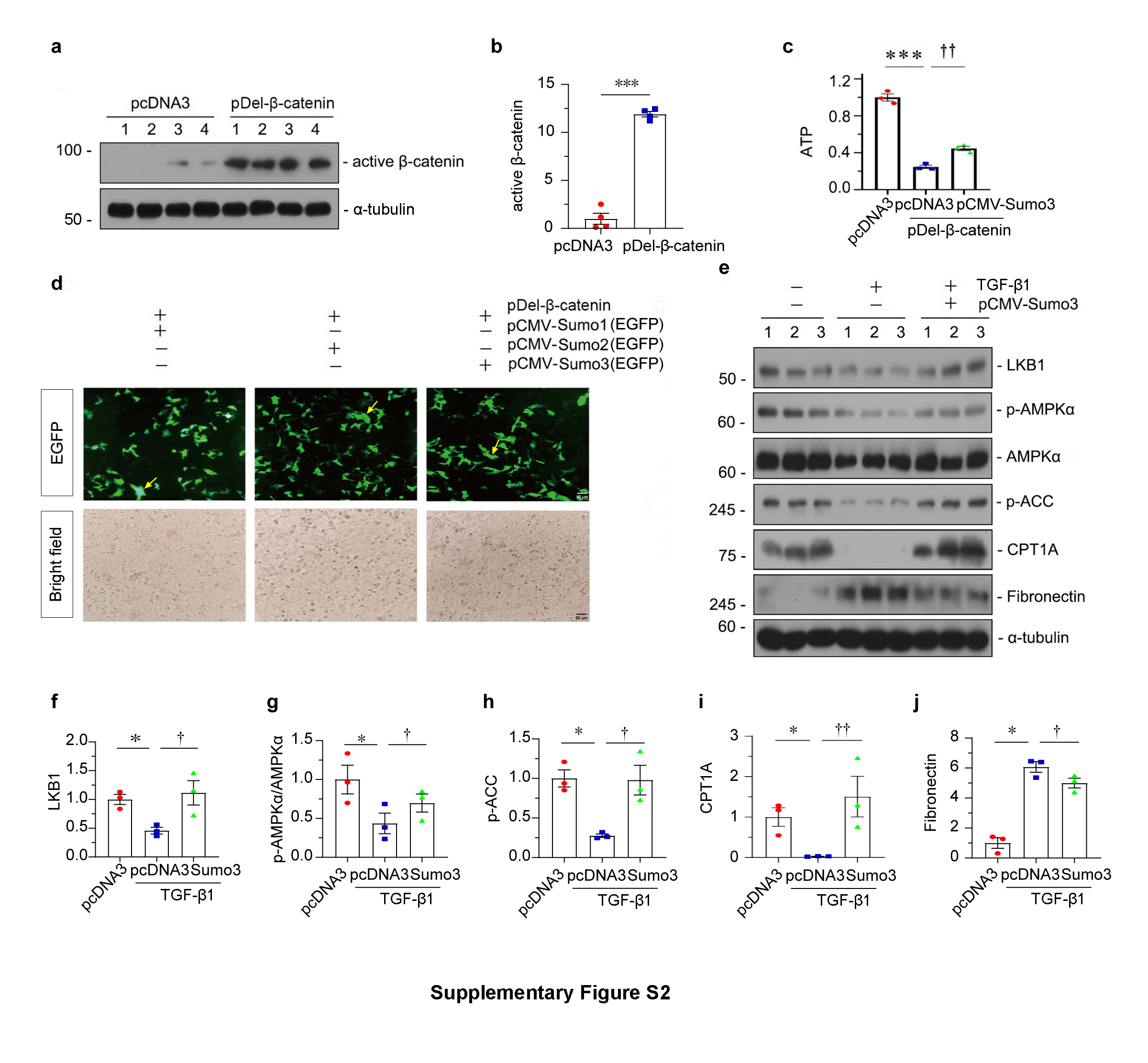

Supplement: Supplementary file 3 — Supplementary Figure S2 [file 41419_2024_7154_MOESM3_ESM.png]

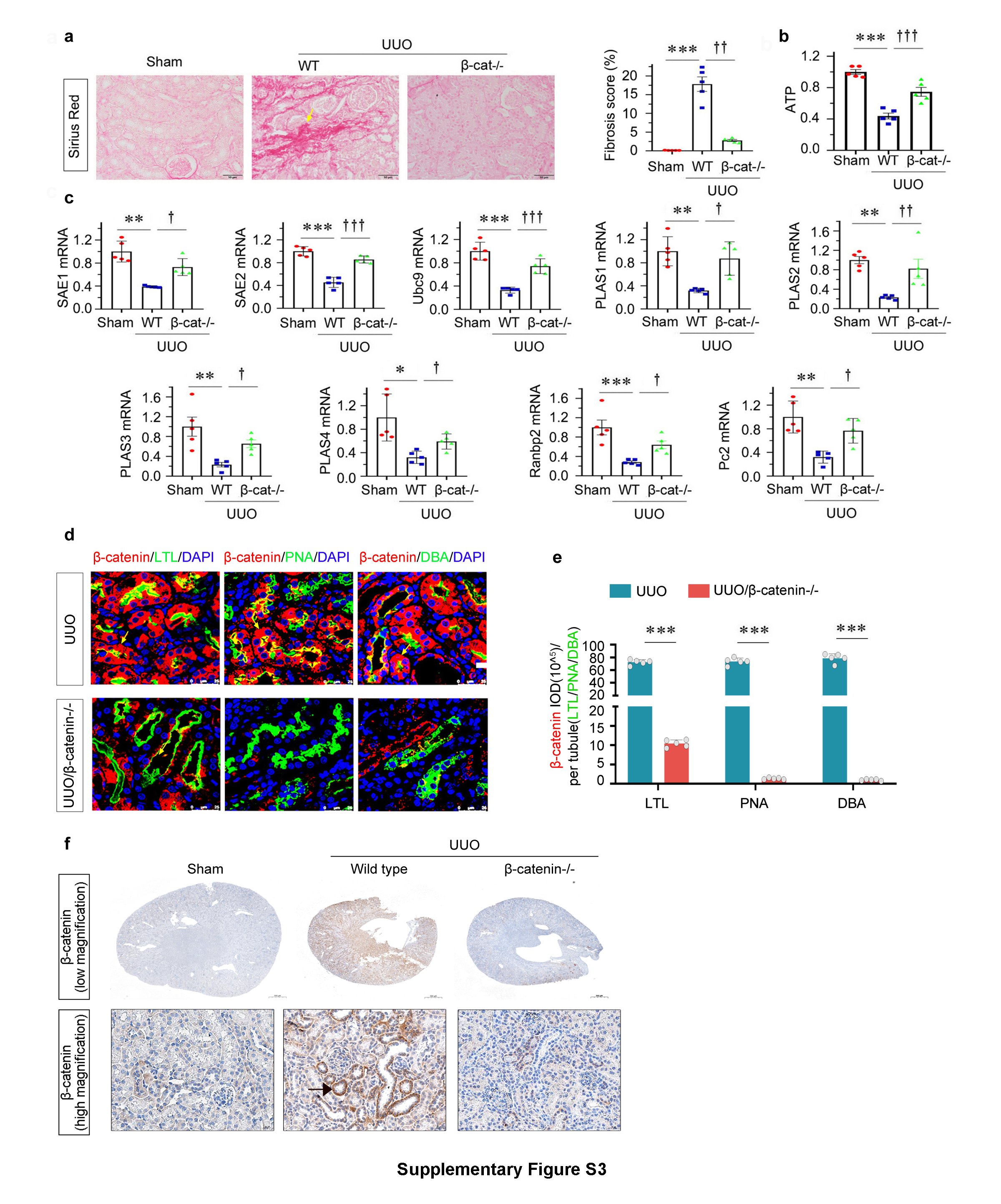

Supplement: Supplementary file 4 — Supplementary Figure S3 [file 41419_2024_7154_MOESM4_ESM.png]
