# Supplementary material for: β-catenin-inhibited Sumoylation modification of LKB1 and fatty acid metabolism is critical in renal fibrosis
Source: Cell Death Dis. 2024 Oct 22;15(10):769. doi: 10.1038/s41419-024-07154-y (PMC11496881; doi:10.1038/s41419-024-07154-y)
Supplement: Supplementary file 5 — Full unedited gels for Western blot [file 41419_2024_7154_MOESM5_ESM.pdf]

Full unedited gels for

β-catenin-inhibited Sumoylation modification of LKB1 and fatty acid metabolism is critical in renal fibrosis

Shuangqin Chen[1,2#], Jiemei Li[1#], Ye Liang[1#], Meijia Zhang[1#], Ziqi Qiu[1#], Sirui Liu[2#], HaoRan Wang[3], Ye Zhu[2], Shicong Song[2], Xiaotao Hou[4], Canzhen Liu[1], Qinyu Wu[1], Mingsheng Zhu[1], Weiwei Shen[1], Jinhua Miao[1], Fan Fan Hou[1], Youhua Liu[1], Cheng Wang[2]*, Lili Zhou[1]*

[#]These authors contributed equally to this work.

*Corresponding author:

Dr. Lili Zhou, Division of Nephrology, Nanfang Hospital, 1838 North Guangzhou Ave, Guangzhou 510515, China, E-mail: jinli730@smu.edu.cn;

Dr. Cheng Wang, Division of Nephrology, Department of medicine, The Fifth Affiliated Hospital Sun Yat-Sen University, Zhuhai, Guangdong 519000, China, E-mail: wangch2@mail.sysu.edu.cn.

Full unedited gels for Figure 1a

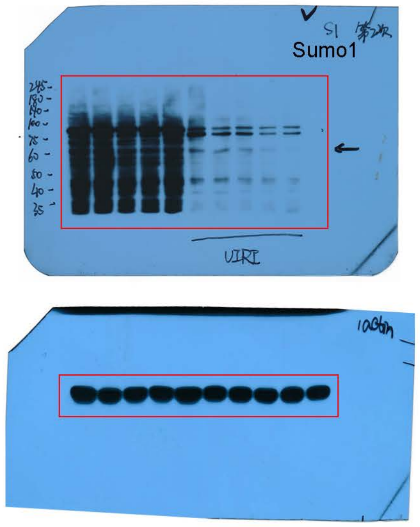

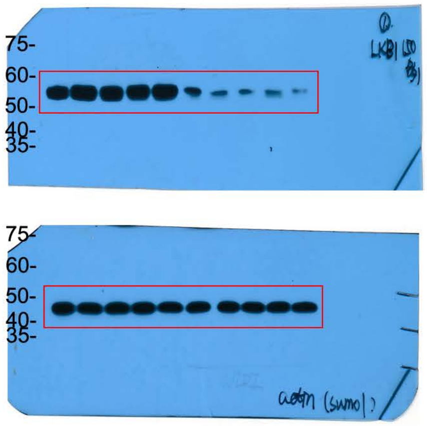

Full unedited gels for Figure 1c

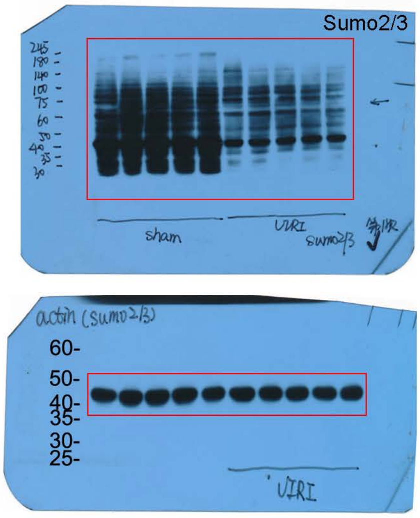

Full unedited gels for Figure 1g

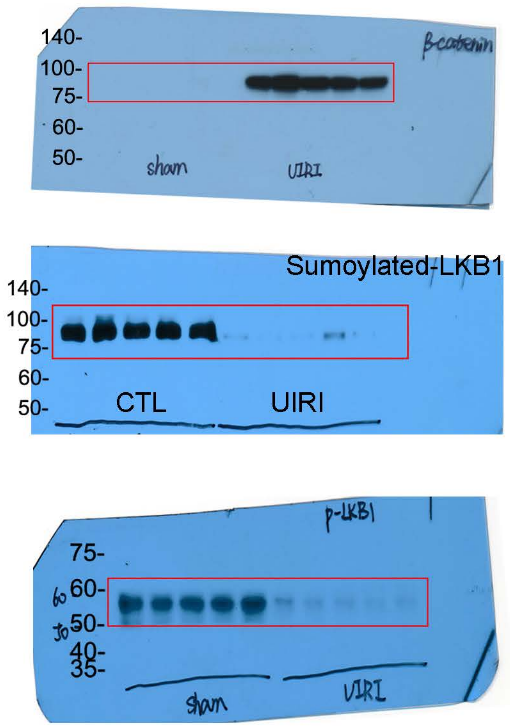

Full unedited gels for Figure 1n

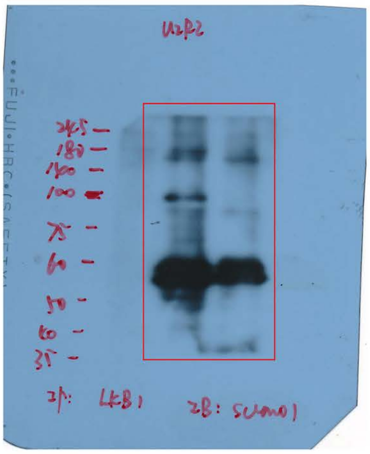

Full unedited gels for Figure 1o

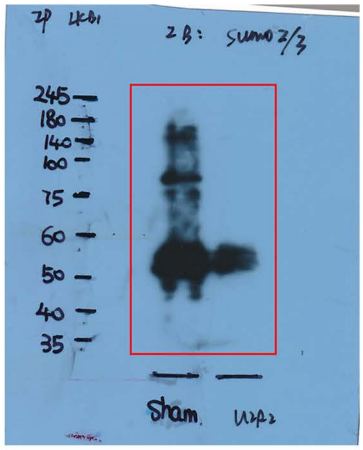

Full unedited gels for Figure 1p

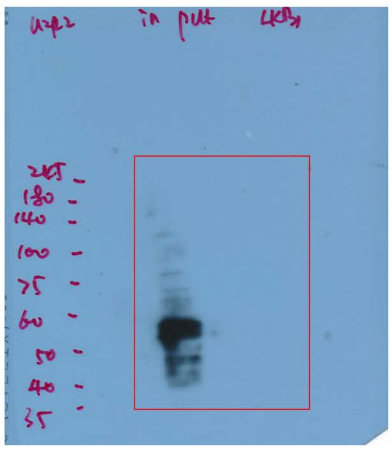

Full unedited gels for Figure 1q

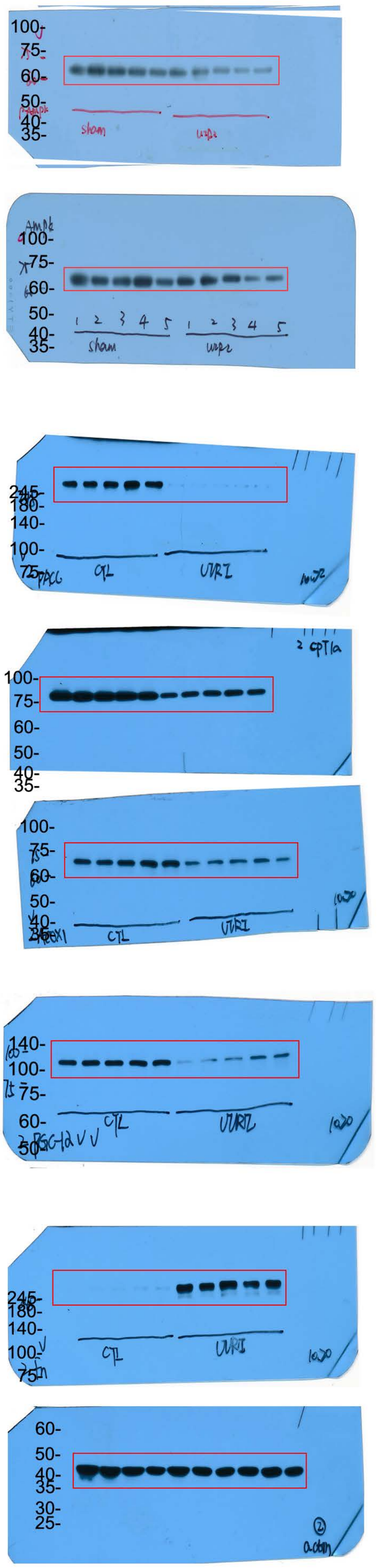

Figure 1

Full unedited gels for Figure 2a

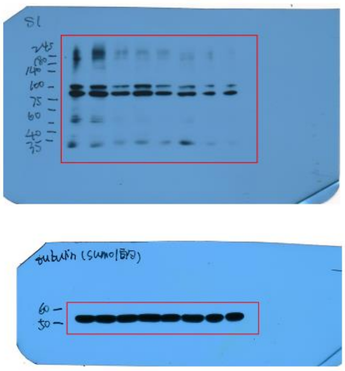

Full unedited gels for Figure 2b

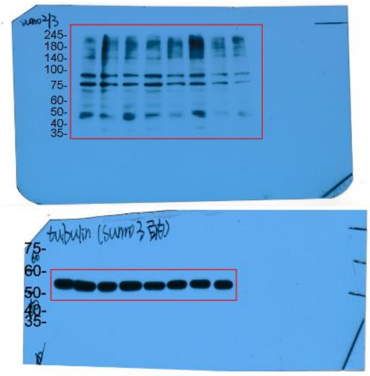

Full unedited gels for Figure 2h

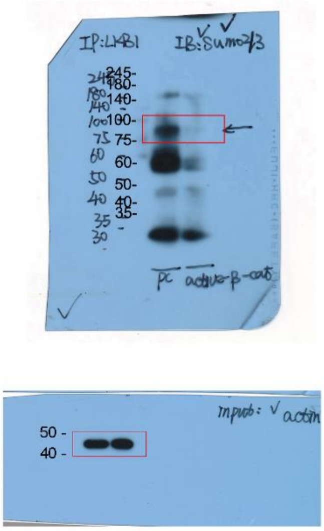

Full unedited gels for Figure 2i

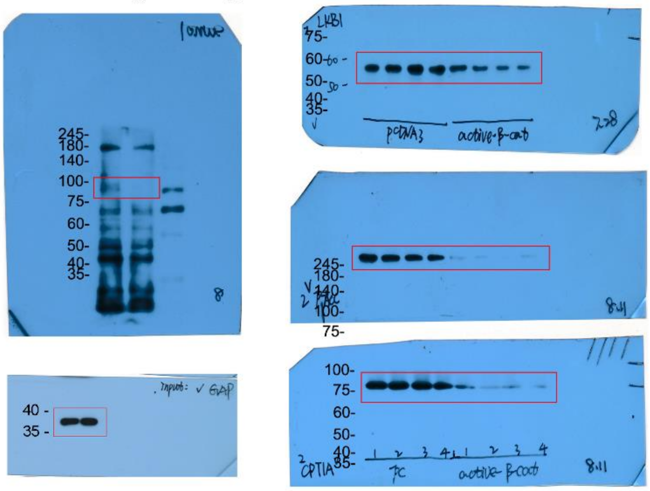

Full unedited gels for Figure 2j

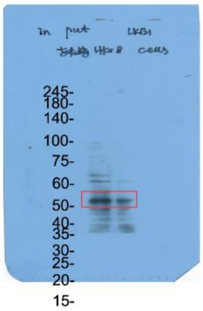

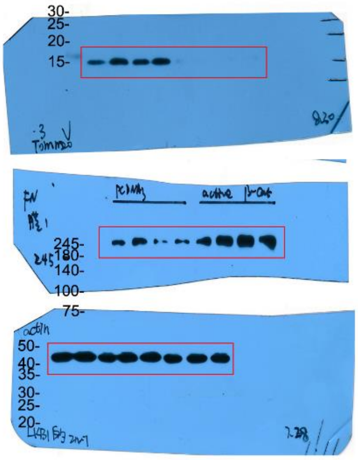

Full unedited gels for Figure 2l

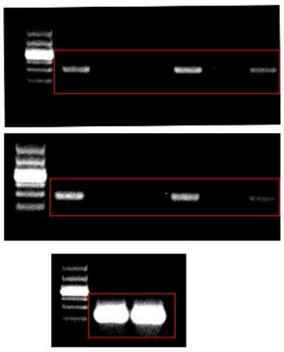

Full unedited gels for Figure 2o

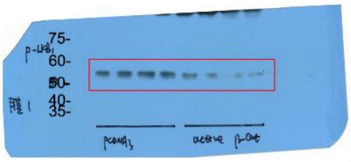

Full unedited gels for Figure 2v

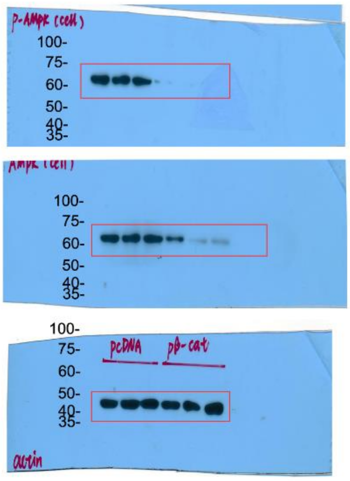

Figure 2

Full unedited gels for Figure 3a

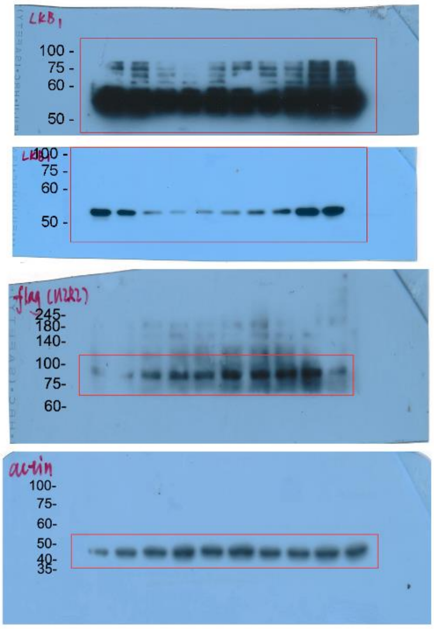

Full unedited gels for Figure 3e

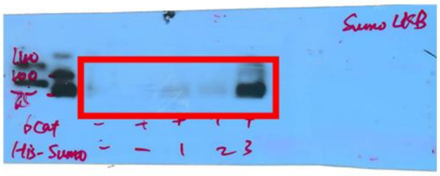

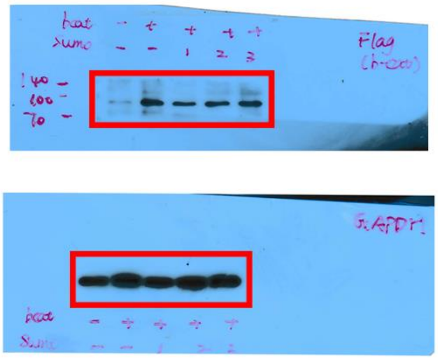

Full unedited gels for Figure 3g

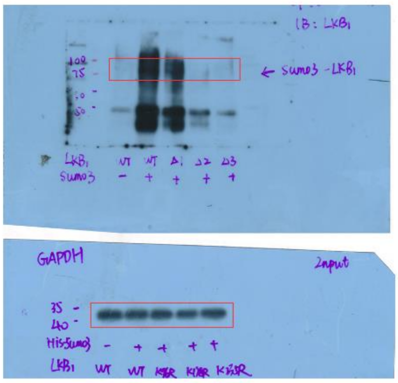

Figure 3

Full unedited gels for Figure 4a

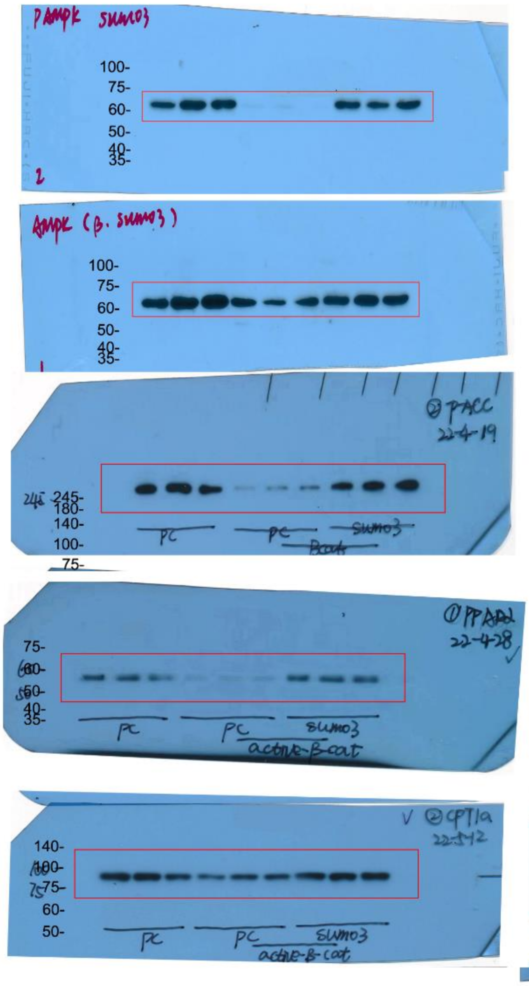

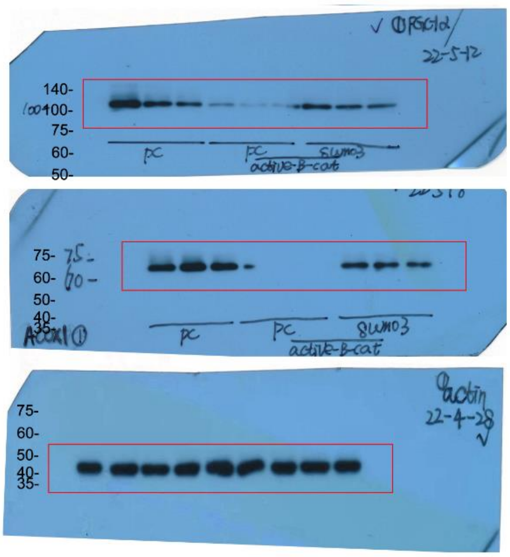

Full unedited gels for Figure 4h

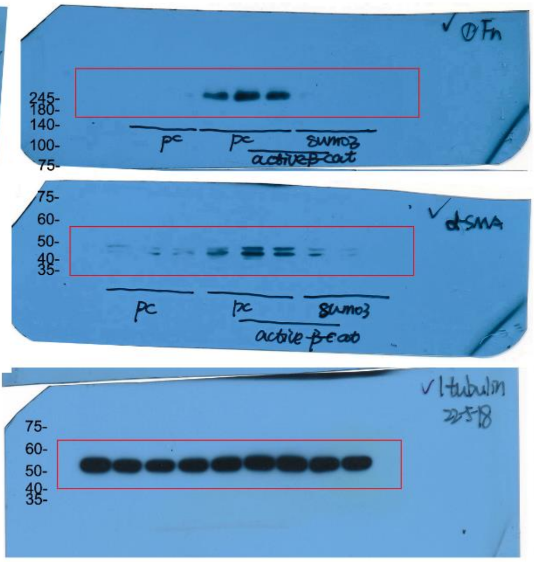

Figure 4

Full unedited gels for Figure 5a

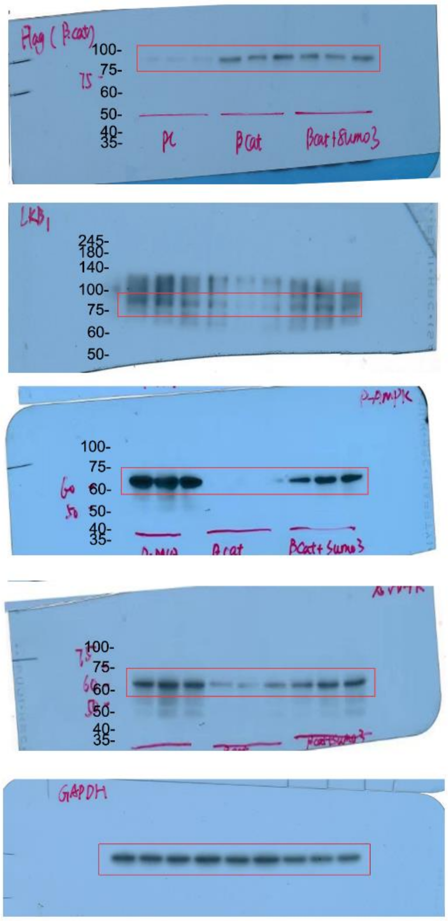

Full unedited gels for Figure 5g

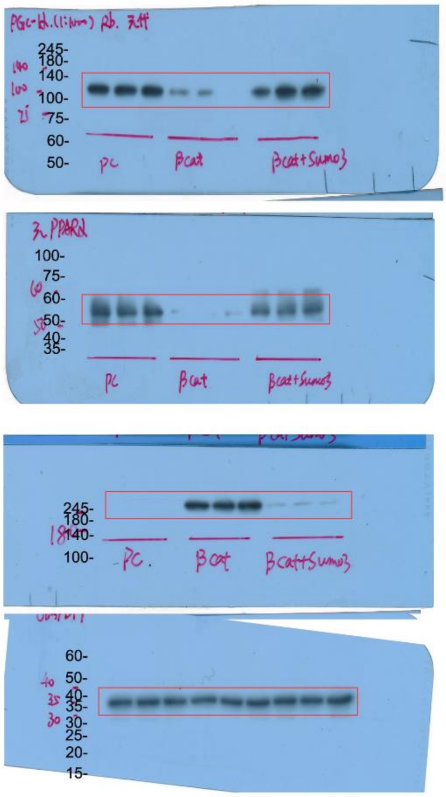

Figure 5

Full unedited gels for Figure 6g

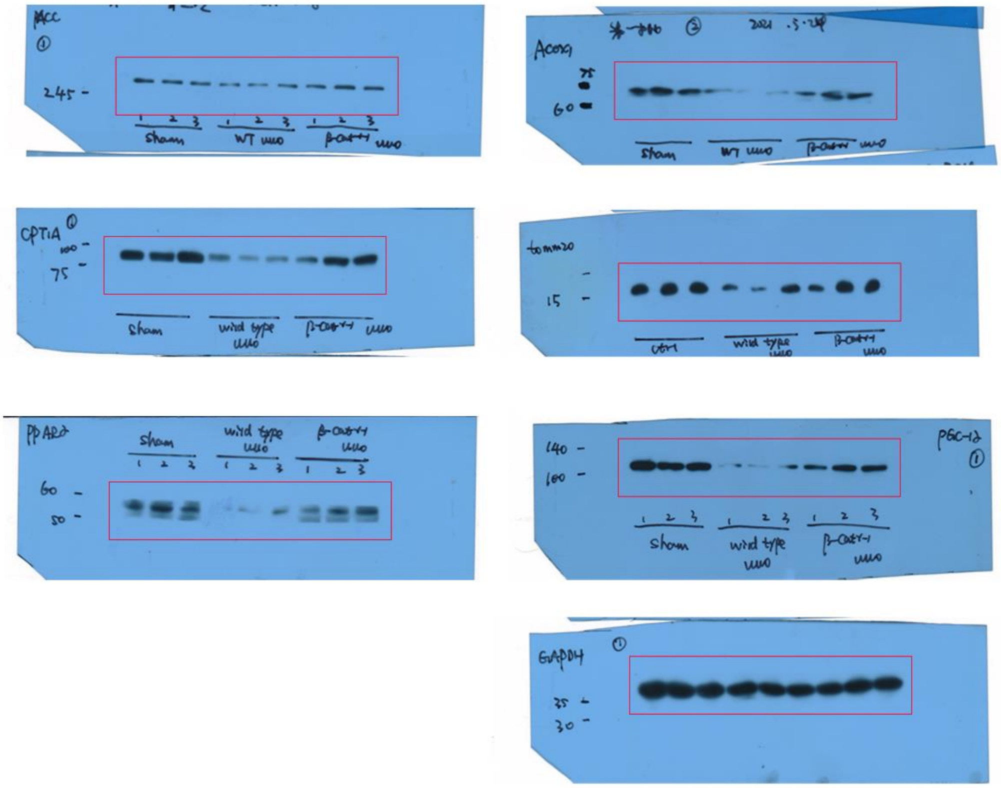

Figure 6

Full unedited gels for Figure 7e

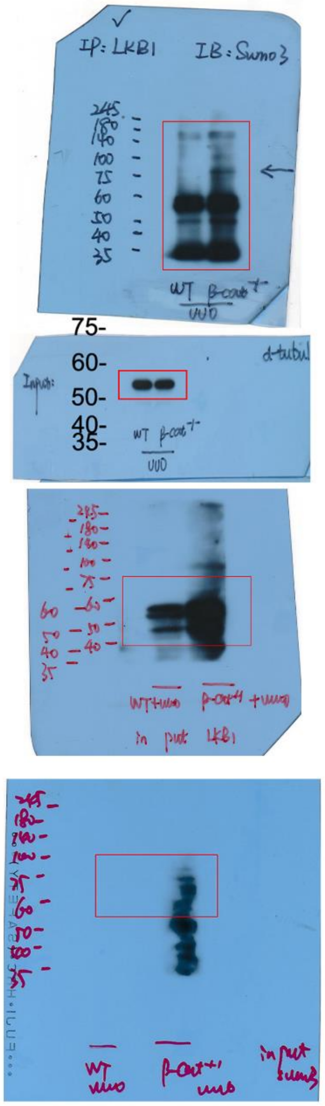

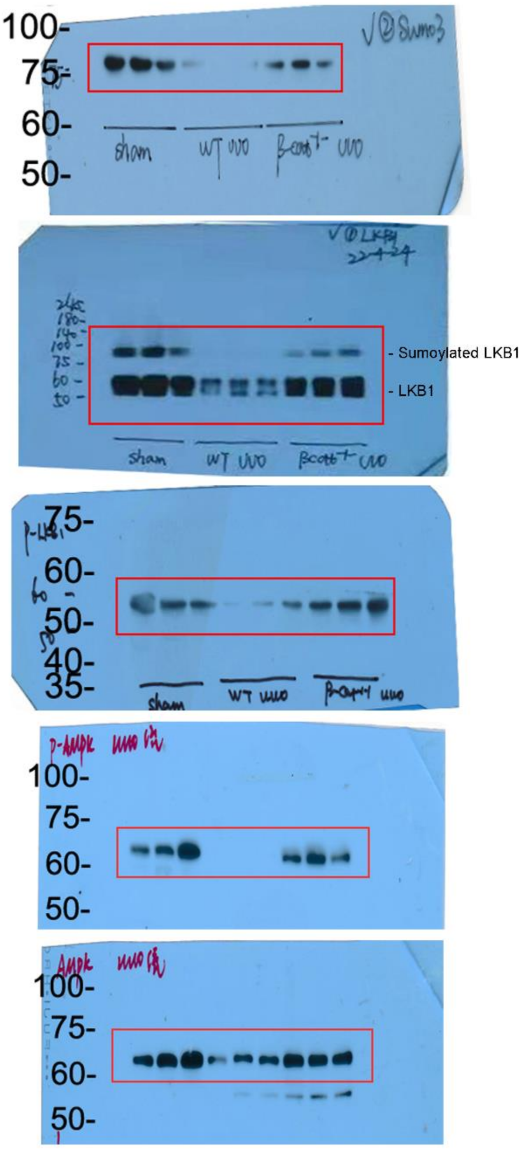

Full unedited gels for Figure 7f

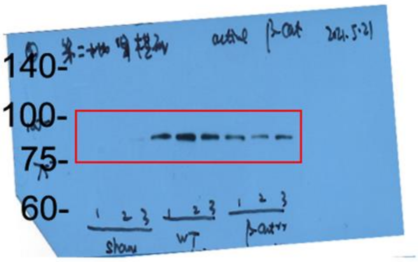

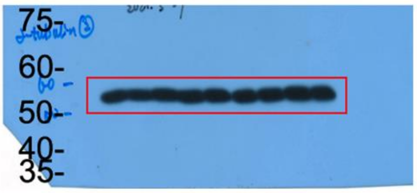

Figure 7

Full unedited gels for Figure 8c

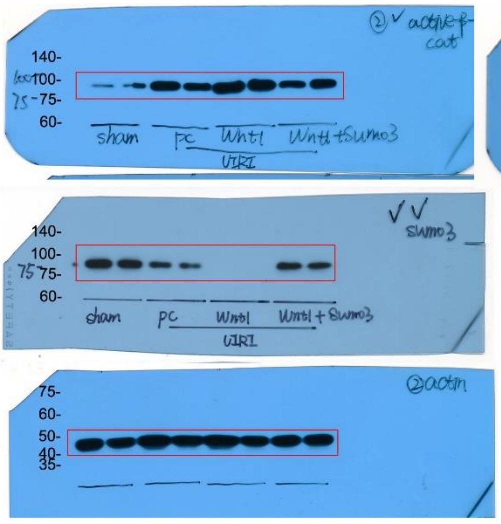

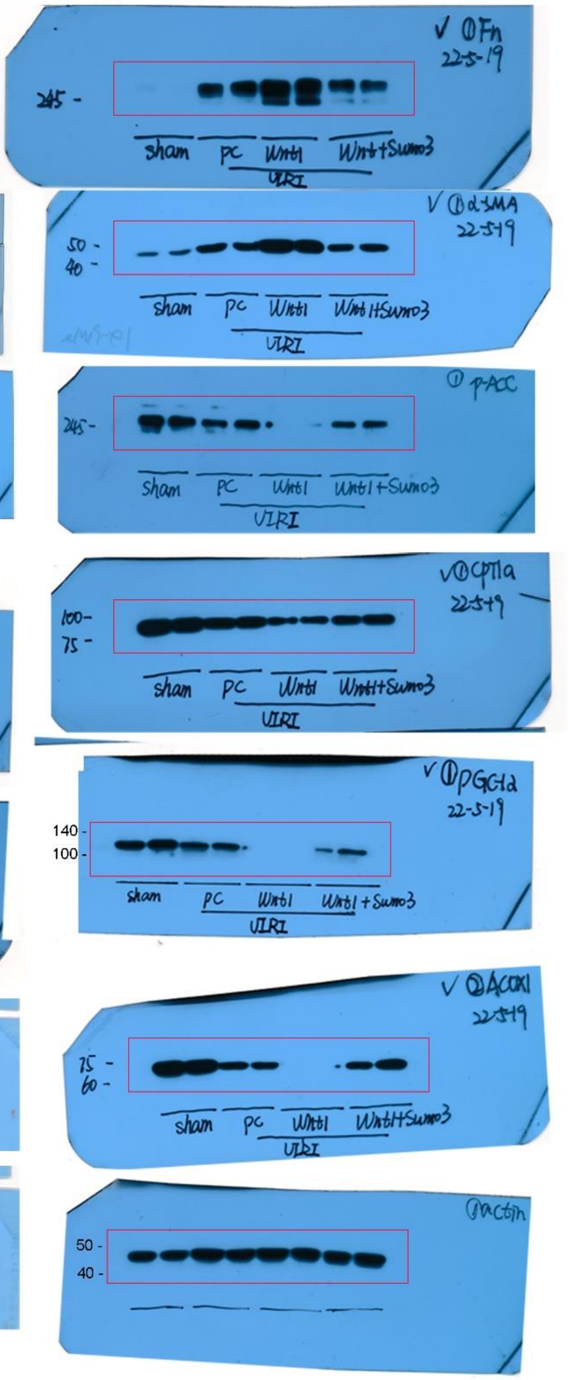

Full unedited gels for Figure 8f

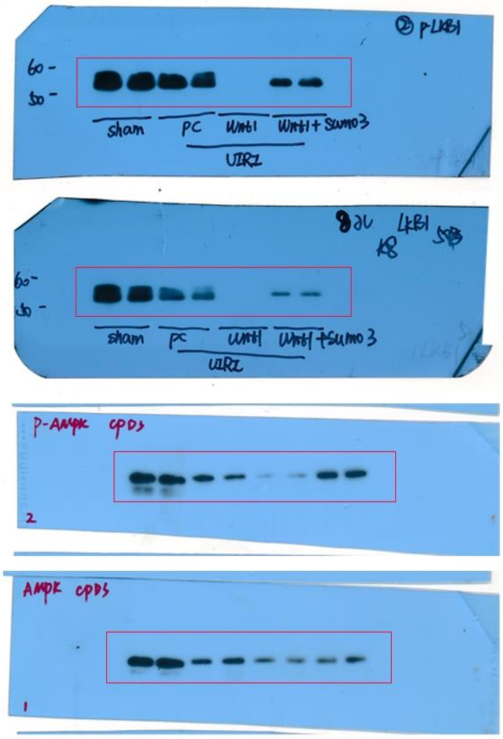

Figure 8

Full unedited gels for Supplementary Figure S2a

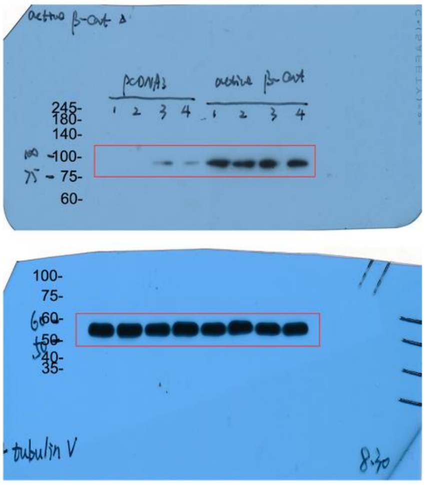

Full unedited gels for Supplementary Figure S2e

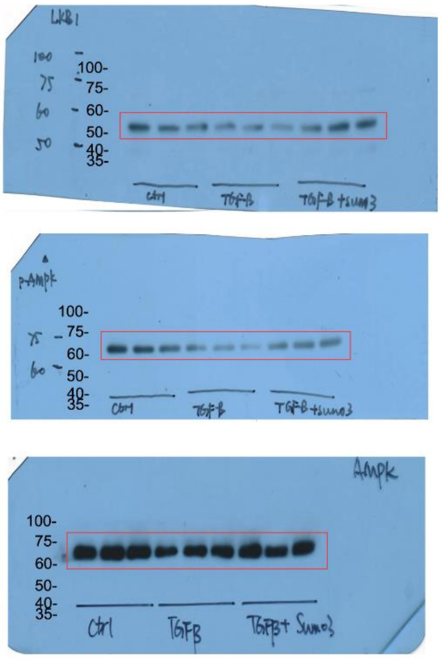

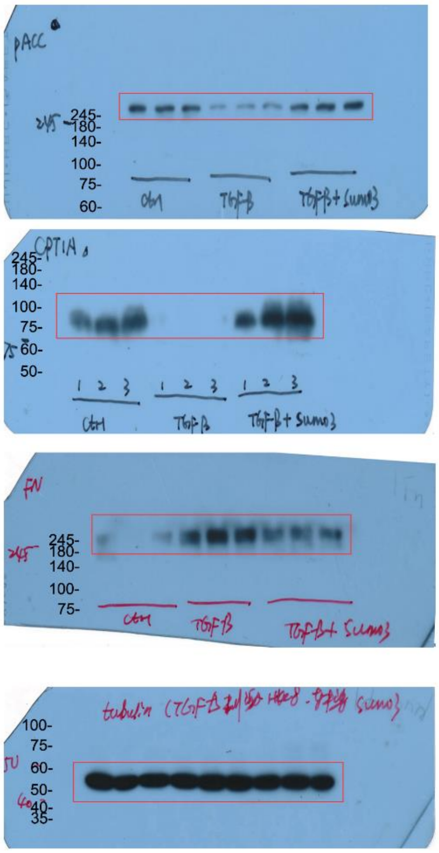

Supplementary Figure S2
